# PRMT5 inhibition suppresses the PI3K/AKT pathway to attenuate vascular smooth muscle cell pathological phenotype in intracranial aneurysm

**DOI:** 10.3389/fneur.2026.1771196

**Published:** 2026-06-18

**Authors:** Junlong Kang, Wei Li, Xinhua Tian, Wei Feng, Xiang Yao, Feng Wei, Hongjin Chen, Junjiang Tong, E. Chen

**Affiliations:** 1 Department of Neurosurgery, Zhongshan Hospital of Xiamen University, School of Medicine, Xiamen University, Xiamen, China; 2 Department of Pharmacy, The Third Hospital of Xiamen, Xiamen, China

**Keywords:** intracranial aneurysm, phenotype switching, PI3K/AKT pathway, protein arginine methyltransferase 5 inhibitor, vascular smooth muscle cells

## Abstract

**Background:**

Protein arginine methyltransferase 5 (PRMT5) regulates vascular smooth muscle cell (VSMC) phenotype switching to participate in cardiovascular disease progression. This study aimed to explore the effect of EPZ015666, a PRMT5 enzymatic activity inhibitor, on VSMC behaviors and phenotype switching in intracranial aneurysm (IA), and potential mechanisms.

**Methods:**

IA rat model was established by ligating left common carotid artery and right renal artery, followed by elastase injection and angiotensin II (Ang II) infusion. Rat brain artery VSMCs were treated with 1 μM Ang II to establish the IA cell model. Then, cells were treated with 10 μM EPZ015666 in combination with Ang II. Furthermore, cells were co-administered with Ang II, EPZ015666, and 50 μg/mL 740Y-P [phosphoinositide 3-kinase (PI3K) agonist].

**Results:**

PRMT5 was increased in the IA specimens of rats and Ang II-treated VSMCs. In Ang II-treated VSMCs, EPZ015666 did not affect PRMT5 but decreased H4R3me2s, suggesting its inhibition of PRMT5 enzymatic activity. EPZ015666 increased VSMC apoptosis, decreased viability, and reduced invasive capacity. EPZ015666 increased *α*-smooth muscle actin and decreased osteopontin and matrix metalloproteinase-9, which indicated that EPZ015666 facilitated the VSMC transition from synthetic phenotype to contractile phenotype. EPZ015666 decreased phosphorylated (p)-PI3K and p-protein kinase B (AKT), suggesting its inhibition of PI3K/AKT pathway. 740Y-P reversed the impact of EPZ015666 on Ang II-treated VSMCs.

**Conclusion:**

EPZ015666 inhibits the PI3K/AKT pathway to promote VSMC phenotype switching from synthetic phenotype to contractile phenotype, thereby attenuating IA progression. However, this study is limited by the use of male-only rats a relatively small sample size, which may restrict the generalizability of the findings.

## Introduction

Intracranial aneurysm (IA) is a pathological dilation of cerebral arteries that may rupture and contribute to subarachnoid hemorrhage (1). Invasive interventions, such as endovascular or surgical therapies, remain the primary management strategies for patients with IA (2–4). However, these invasive interventions may lead to additional complications in patients with IA (5, 6). Therefore, exploring potential noninvasive interventions is vital in improving the management of patients with IA.

Vascular smooth muscle cells (VSMCs) play an essential role in maintaining vascular integrity, and their phenotype switching is closely involved in IA pathogenesis (7, 8). The phenotype switching refers to the transition of VSMCs from a contractile phenotype to a synthetic phenotype in response to injury (7). Several previous studies have disclosed that the phenotypic modulation of VSMCs could alter IA formation and progression (9–13).

Protein arginine methyltransferase 5 (PRMT5), an epigenetic regulator, modulates gene expression and cellular function by catalyzing the methylation of proteins (14, 15). Some previous studies have found that PRMT5 is involved in the pathogenesis and progression of cardiovascular diseases (16–19). Importantly, a previous study discovered that PRMT5 inhibition could suppress proliferation, reduce migration, and promote phenotypic switching from a synthetic to a contractile state in VSMCs (20).

EPZ015666, an inhibitor of PRMT5 enzymatic activity, possesses a protective role in cardiovascular diseases (19–22). For example, a previous study reported that EPZ015666 inhibited cardiac fibroblast activation *in vitro* (22). Moreover, EPZ015666 suppressed both cardiac hypertrophy and fibrosis *in vivo* (19). Of note, a previous study showed that PRMT5 regulated VSMC phenotypic switching and vascular remodeling in the context of proliferative vascular diseases, and the pharmacological inhibition of PRMT5 with EPZ015666 attenuated carotid artery ligation-induced neointimal formation (20). However, this previous study focused on proliferative vascular disease model. The role of PRMT5 inhibition by EPZ015666 in IA, particularly its effect on VSMC dysfunction under Ang II stimulation and the underlying mechanisms, has not been specifically investigated.

Accordingly, this study aimed to explore the effect of EPZ015666 on VSMC behaviors and phenotype switching in IA, and the potential mechanisms.

## Materials and methods

### IA rat model

Sprague Dawley rats (8 weeks old, SLAC, China) were adopted to construct the IA rat model using modified methods according to previous studies (23–25). Briefly, after being anesthetized by inhalation of 2% isoflurane (MCE, China), the left common carotid artery and right renal artery were ligated, followed by injection of 10 μL of elastase (10 U/mL, MCE, China) into the right basal cistern on Day 7. Following that, osmotic pumps (ALZET, United States) containing angiotensin II (Ang II) (500 ng/kg/min, MCE, China) were implanted into subcutaneous pockets in the dorsal skin between the scapulae of rats for 28 days. The rats in the Model group (*n* = 5) were euthanized on Day 35 by gradual-fill carbon dioxide inhalation at a displacement rate of 40% of the chamber volume per minute, and the IA specimens were harvested. The rats in the Sham group (*n* = 5) only underwent surgical incisions without ligation, elastase injection, and osmotic pump implantation, followed by euthanasia on Day 35 using the same carbon dioxide inhalation method, and the normal brain artery specimens were harvested. Cerebral arteries were visualized by perfusion with a gelatin solution containing bromophenol blue dye before euthanasia. Tissue harvesting was performed by investigators blinded to experimental group allocation. Animal protocols were approved by the Animal Care and Use Committee and followed the relevant guidelines.

### Immunohistochemistry (IHC) staining

IA specimens (Model group) and normal brain artery specimens (Sham group) were fixed, embedded, and cut into sections. After being dewaxed, rehydrated, antigen-repaired, and blocked, sections were exposed to anti-PRMT5 antibody (1:100; Cat. no. DF6439; Affinity, China) or anti-phosphorylated (p)-phosphoinositide 3-kinase (PI3K) (Tyr458) (1:100; Cat. no. AF3242; Affinity, China) overnight, followed by incubation with secondary antibody (1:1000; Servicebio, China) for 1 h and staining with DAB Kit (Servicebio, China). The IHC score was performed based on staining intensity (0, negative; 1, weak; 2, moderate; 3, strong) and the proportion of positive cells (0, <5%; 1, 5–25%; 2, 26–50%; 3, 51–75%; 4, >75%), and the final IHC score was obtained by multiplying the two scores. IHC score was independently performed by two blinded investigators, and the averaged value was used for statistical analysis.

### Cell culture

Rat brain artery VSMCs were obtained from Procell (Wuhan, China). Cells were cultivated using Dulbecco’s Modified Eagle Medium (Servicebio, China) plus 10% fetal bovine serum (Servicebio, China). Subculturing was implemented via trypsin (Servicebio, China) when reaching 90% confluence, and cells at passages 3–7 were utilized for subsequent experiments.

### IA cell model

To establish IA cell model *in vitro*, cells were subjected to 24-h stimulation with 0, 0.1, 0.5, or 1 μM Ang II, and served as the Untreated group, Ang II (0.1 μM) group, Ang II (0.5 μM) group, and Ang II (1 μM) group, respectively. Cells were adopted for the detection of cell viability. Meanwhile, the protein expression of PRMT5 and PI3K pathway was assessed with a Western blotting assay. Based on our results and a previous study (26), the Ang II at a concentration of 1 μM was used to establish the IA cell model.

### EPZ015666 treatment

EPZ015666 (MCE, China) is an orally bioavailable small-molecule inhibitor of PRMT5 enzymatic activity. Cells were treated with EPZ015666 (0, 1, 5, or 10 μM) in combination with Ang II (1 μM) for 24 h, then set as the Model group, EPZ (1 μM) group, EPZ (5 μM) group, and EPZ (10 μM) group, respectively. Following that, cells were adopted for cell viability, apoptosis, invasive capacity, and Western blotting assays. Based on the findings across different EPZ015666 concentrations (1, 5, and 10 μM) and the calculated half-maximal inhibitory concentration (IC_50_) for H4R3me2s inhibition (3.850 ± 0.785 μM), 10 μM EPZ015666 (approximately 2.6-fold the IC_50_) was selected to ensure robust inhibition of PRMT5 enzymatic activity while minimizing potential off-target effects, and was used in the subsequent 740Y-P treatment experiment.

### 740Y-P treatment

740Y-P (MCE, China), a PI3K agonist, was utilized to reactivate the PI3K-protein kinase B (AKT) pathway in EPZ015666-treated cells. Briefly, cells were divided into four groups. The cells in the Model group were stimulated with 1 μM Ang II. The cells in the EPZ group were treated with 1 μM Ang II and 10 μM EPZ015666. The cells in the 740Y-P group were given treatment of 1 μM Ang II and 50 μg/mL 740Y-P (27). The cells in the EPZ + 740Y-P group were co-administered with 1 μM Ang II, 10 μM EPZ015666, and 50 μg/mL 740Y-P. After 24 h of treatment, cells were adopted for cell viability, apoptosis, invasive capacity, and Western blotting assays. The concentration of 740Y-P used (50 μg/mL, approximately 15 μM) was below the threshold (100 μM) for nonspecific membrane toxicity.

### Cell viability

Cell viability was assessed with Cell Counting Kit-8 (Servicebio, China). In brief, cells were replated and cultivated for 24 h. Cells were then added with 10% detection reagent for 2 h, and optical density (OD) was assessed using a microplate reader (Huadong, China).

### Western blotting

The proteins were isolated using RIPA Lysis Buffer (Servicebio, China) in the presence of protease inhibitors (Servicebio, China). Protein concentrations were determined via the Bicinchoninic Acid Kit (Servicebio, China). Equal amounts were separated using the 4–20% SDS-PAGE Precast Gel (Willget, China) and transferred to BioTrace Nitrocellulose Membrane (Pall, United States). Membranes were exposed to the blocking solution (Servicebio, China), followed by treatment with primary antibodies, including PRMT5 (Cat. no. GB112970; Servicebio, China), H4R3me2s (Cat. no. 61187; Proteintech, China), Histone H4 (Cat. no. 16047-1-AP; Proteintech, China), *α*-smooth muscle actin (SMA) (Cat. no. GB111364; Servicebio, China), osteopontin (OPN) (Cat. no. GB120018; Servicebio, China), matrix metalloproteinase-9 (MMP9) (Cat. no. Affinity, China), p-PI3K (Tyr458) (Cat. no. AF3242; Affinity, China), PI3K (Cat. No. AF6241; Affinity, China), p-AKT (Ser473) (Cat. no. 28731-1-AP; Proteintech, China), AKT (Cat. no. 10176-2-AP; Proteintech, China), Cleaved caspase-3 (Cat. no. AF7022; Affinity, China) and GAPDH (Cat. no. AF7021; Affinity, China), at recommended dilution. After that, membranes were exposed to secondary antibodies (1:20000) (Cat. no. GB23303; Servicebio, China) and detected using Super ECL Kit (Servicebio, China). Band intensities were quantified with ImageJ (V1.8; NIH, United States), and quantification was not conducted under blinded conditions.

### Cell apoptosis

Cell apoptosis assay was carried out via Annexin V-FITC Apoptosis Kit (Beyotime, China). Briefly, cells were harvested and resuspended in binding buffer comprising Annexin V-FITC and propidium iodide (PI) (Beyotime, China). Flow cytometric quantification was carried out, and data were analyzed by FlowJo (V10; BD, United States).

### Cell invasive capacity

Matrigel-coated Transwell chambers (Corning, China) were employed for the detection of cell invasive capacity. Serum-starved cells were seeded in the upper chambers, with complete medium in the lower chambers. Following 24 h culture, invasive cells were treated with 4% paraformaldehyde and stained by crystal violet (Servicebio, China). Cells were counted under a microscope (Motic, China). To minimize the potential influence of cell proliferation and apoptosis on invasive capacity results, additional Transwell assays were performed with a shortened incubation time of 6 h.

### Statistical analysis

Statistical analyses were accomplished via GraphPad Prism 9 (GraphPad Software, United States). Data were shown as mean ± standard deviation (SD). For comparisons between two groups, an unpaired t test was used. A one-way analysis of variance (ANOVA) was performed, followed by Tukey’s *post hoc* tests, to determine statistical differences among multiple groups. Prior to parametric analyses, data distribution normality was assessed using the Shapiro–Wilk test. Homogeneity of variance was evaluated using Brown-Forsythe test. Incidence of IA was analyzed using Fisher’s exact test. *p* < 0.05 was considered statistically significant.

## Results

### PRMT5 and p-PI3K levels in IA rat and cell models

Latex perfusion revealed an evident aneurysmal dilation in the Model group compared to the Sham group, and the incidence of IA in the Model group was significantly higher than in the Sham group (80.0% vs. 0.0%) (*p* < 0.05) (Supplementary Figure 1).

**Figure 1:**
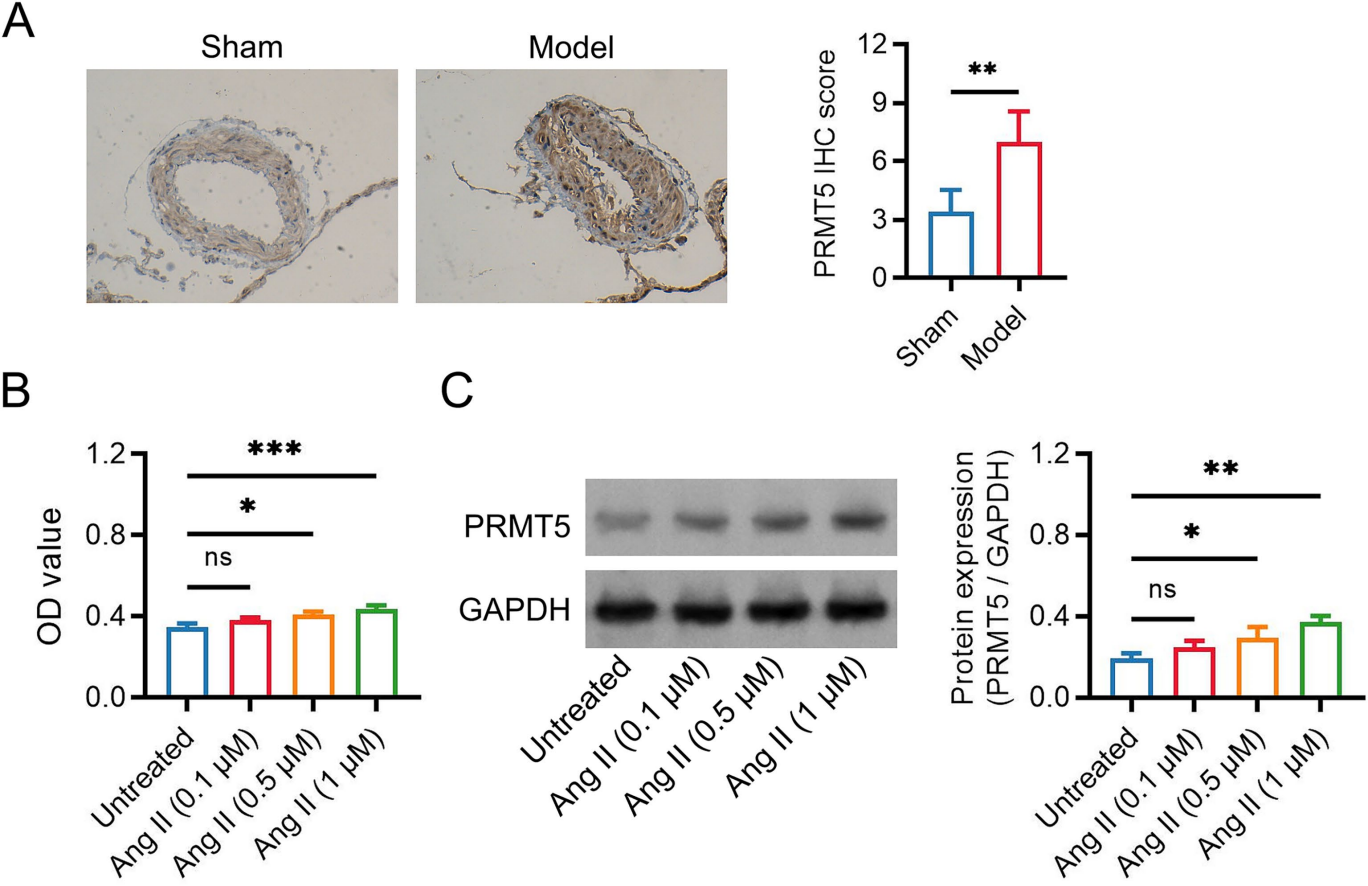
PRMT5 level was increased in IA models. *In vivo* analysis of PRMT5 level between the sham and model groups **(A)**. *In vitro* analysis of VSMC viability **(B)** and PRMT5 level **(C)** among the untreated, Ang II (0.1 μM), Ang II (0.5 μM), and Ang II (1 μM) groups. *: *p* < 0.05; **: *p* < 0.01; ***: *p* < 0.001; ns: not significant.

In animal experiments, the PRMT5 (Figure 1A) and p-PI3K (Supplementary Figure 2A) levels were higher in the IA specimens of the Model group than in the normal brain artery specimens of the Sham group (both *p* < 0.05). In cell experiments, the VSMC viability was increased in Ang II (0.5 μM) (*p* < 0.05) and Ang II (1 μM) (*p* < 0.001) groups compared to the Untreated group (Figure 1B). PRMT5 (Figure 1C) and p-PI3K (Supplementary Figure 2B) levels were elevated in Ang II (0.5 μM) and Ang II (1 μM) groups compared to the Untreated group (all *p* < 0.05). Overall, PRMT5 had a high level in the IA specimens of rats and Ang II-treated VSMCs.

### Effect of EPZ015666 on apoptosis, viability, and invasive capacity in the IA cell model

**Figure 2:**
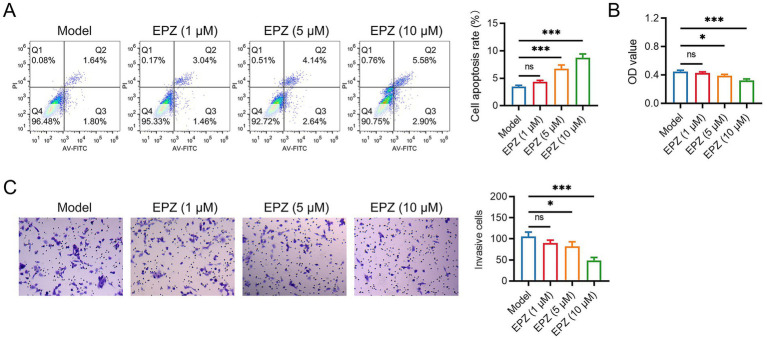
EPZ015666 promoted apoptosis, reduced viability, and attenuated invasive capacity in Ang II-treated VSMCs. Comparison of VSMC apoptosis **(A)**, viability **(B)**, and invasive capacity **(C)** among the model, EPZ (1 μM), EPZ (5 μM), and EPZ (10 μM) groups. *: *p* < 0.05; ***: *p* < 0.001; ns: not significant.

VSMC apoptosis was increased in the EPZ (5 μM) (*p* < 0.001) and EPZ (10 μM) (*p* < 0.001) groups compared to the Model group (Figure 2A). VSMC viability (Figure 2B) and invasive capacity (Figure 2C) were decreased in the EPZ (5 μM) and EPZ (10 μM) groups compared to the Model group (all *p* < 0.05). The above findings indicated that EPZ015666 enhanced VSMC apoptosis, reduced viability, and reduced invasive capacity.

### Effect of EPZ015666 on PRMT5 enzymatic activity, phenotype markers, and the PI3K/AKT pathway in the IA cell model

**Figure 3:**
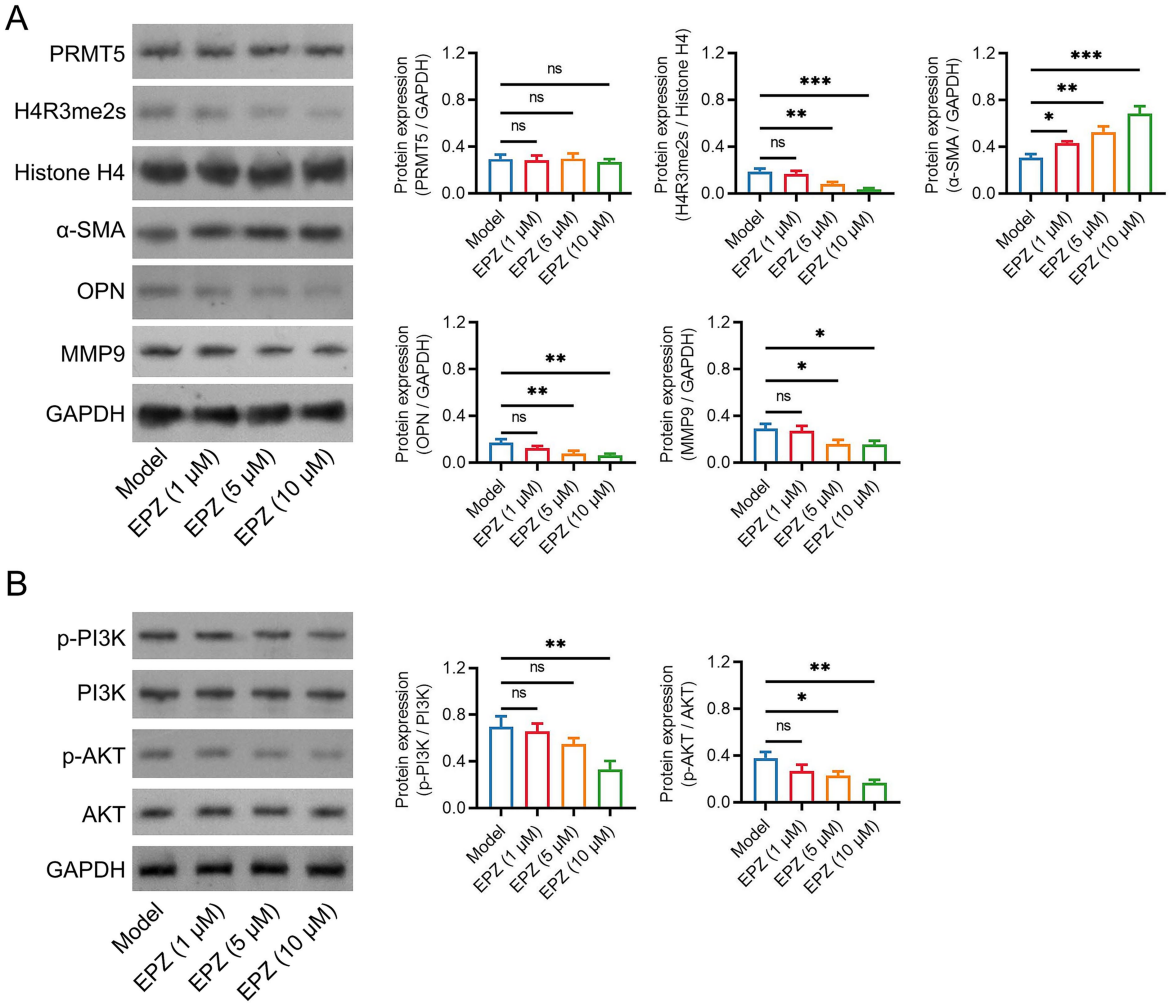
EPZ015666 inhibited PRMT5 enzymatic activity, facilitated transition from synthetic phenotype to contractile phenotype, and suppressed PI3K/AKT pathway in Ang II-treated VSMCs. Comparison of PRMT5, H4R3me2s, *α*-SMA, OPN, and MMP9 levels among the Model, EPZ (1 μM), EPZ (5 μM), and EPZ (10 μM) groups **(A)**. Comparison of p-PI3K and p-AKT among the Model, EPZ (1 μM), EPZ (5 μM), and EPZ (10 μM) groups **(B)**. *: *p* < 0.05; **: *p* < 0.01; ***: *p* < 0.001; ns: not significant.

PRMT5 protein level was not different between EPZ (1, 5, or 10 μM) and Model groups (all *p* > 0.05). However, H4R3me2s protein level was decreased in the EPZ (5 μM) (*p* < 0.01) and EPZ (10 μM) (*p* < 0.001) groups compared to the Model group (Figure 3A). The IC_50_ of EPZ for H4R3me2s inhibition was calculated as 3.850 ± 0.785 μM (Supplementary Figure 3). These findings indicated that EPZ015666 did not affect the PRMT5 level but reduced its enzymatic activity.

Regarding VSMC phenotype markers, *α*-SMA protein level was increased in all EPZ (1, 5, or 10 μM) groups compared to the Model group (all *p* < 0.05). OPN and MMP9 protein levels were decreased in the EPZ (5 μM) and EPZ (10 μM) groups compared to the Model group (all *p* < 0.05) (Figure 3A). The above findings suggested that EPZ015666 promoted VSMC transition from the synthetic phenotype to the contractile phenotype.

Regarding the PI3K/AKT pathway, p-PI3K protein level was decreased in the EPZ (10 μM) group compared to the Model group (*p* < 0.01); p-AKT protein level was reduced in the EPZ (5 μM) (*p* < 0.05) and EPZ (10 μM) (*p* < 0.01) groups compared to the Model group (Figure 3B). Therefore, EPZ015666 inhibited the PI3K/AKT pathway.

### Effect of 740Y-P on PI3K/AKT pathway, viability, apoptosis, invasive capacity, and phenotype switching in the IA cell model under EPZ015666 treatment

**Figure 4:**
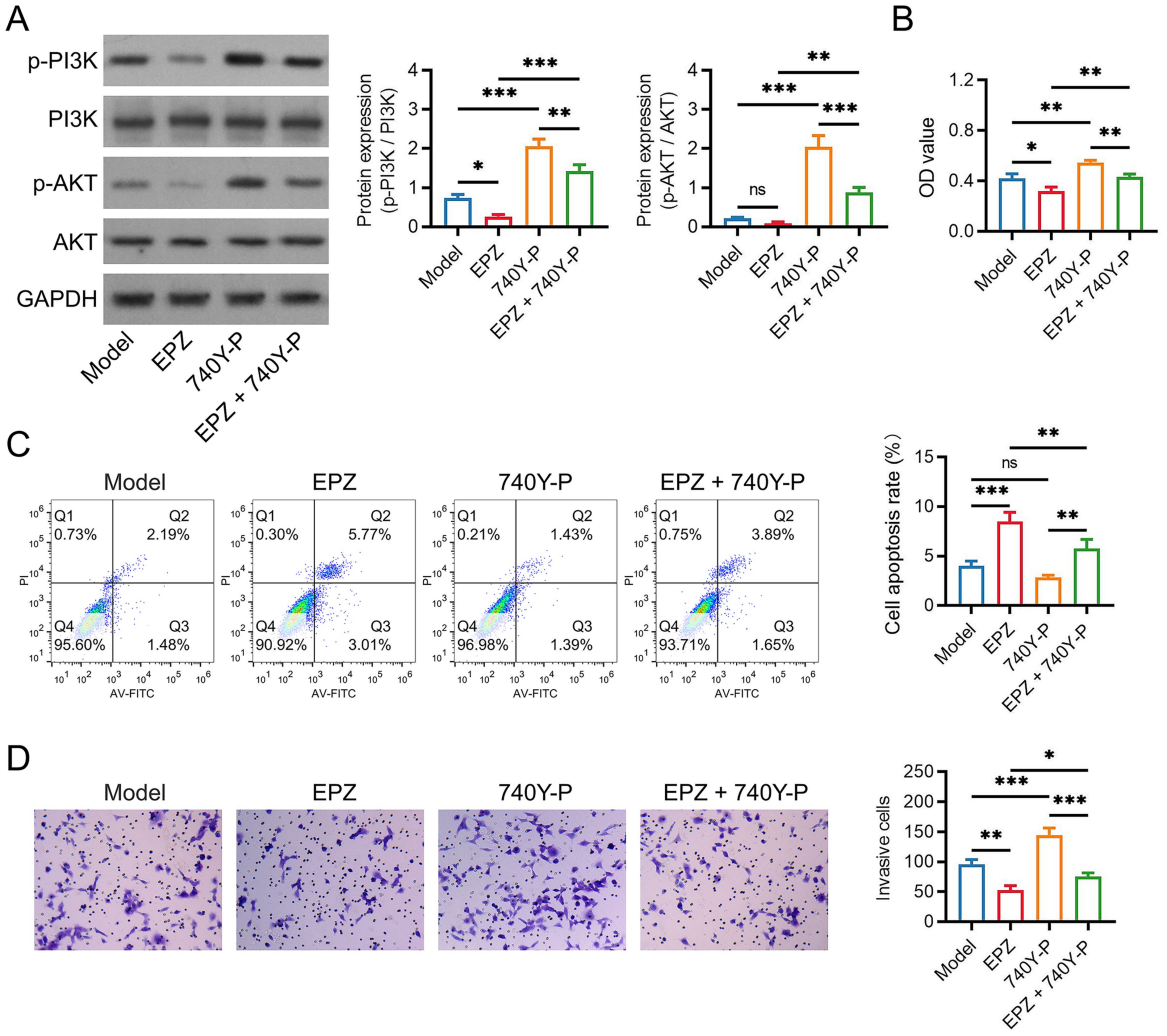
740Y-P reversed the effect of EPZ015666 on viability, apoptosis, invasive capacity, and PI3K/AKT pathway in Ang II-treated VSMCs. Comparison of p-PI3K and p-AKT levels **(A)**, viability **(B)**, apoptosis **(C)**, and invasive capacity **(D)** among the model, EPZ, 740Y-P, and EPZ + 740Y-P groups. *: *p* < 0.05; **: *p* < 0.01; ***: *p* < 0.001; ns: not significant.

The p-PI3K and p-AKT protein levels were increased in the 740Y-P group vs. the Model group (both *p* < 0.001), and in the EPZ + 740Y-P group vs. the EPZ group (both *p* < 0.01) (Figure 4A). These findings indicated that 740Y-P activated the PI3K/AKT pathway and reversed the effect of EPZ015666 on this pathway.

VSMC viability was elevated in the 740Y-P group vs. the Model group (*p* < 0.01), and in the EPZ + 740Y-P group vs. the EPZ group (*p* < 0.01) (Figure 4B). VSMC apoptosis was decreased in the EPZ + 740Y-P group vs. the EPZ group (*p* < 0.01) (Figure 4C). Notably, cleaved caspase-3 expression showed a consistent trend with the flow cytometry results, further confirming the effect of EPZ015666 on VSMC apoptosis and the involvement of the PI3K/AKT pathway (Supplementary Figure 4A). VSMC invasive capacity was enhanced in the 740Y-P group vs. the Model group (*p* < 0.001), and in the EPZ + 740Y-P group vs. the EPZ group (*p* < 0.05) (Figure 4D). Moreover, additional Transwell assays performed with a shortened incubation time (6 h) yielded consistent results, suggesting that the observed differences in invasive capacity were not attributable to proliferation bias (Supplementary Figure 4B). Overall, 740Y-P enhanced VSMC viability, reduced apoptosis, and increased invasive capacity. Meanwhile, 740Y-P partially reversed the effect of EPZ015666 on VSMC viability, apoptosis, invasive capacity.

**Figure 5:**
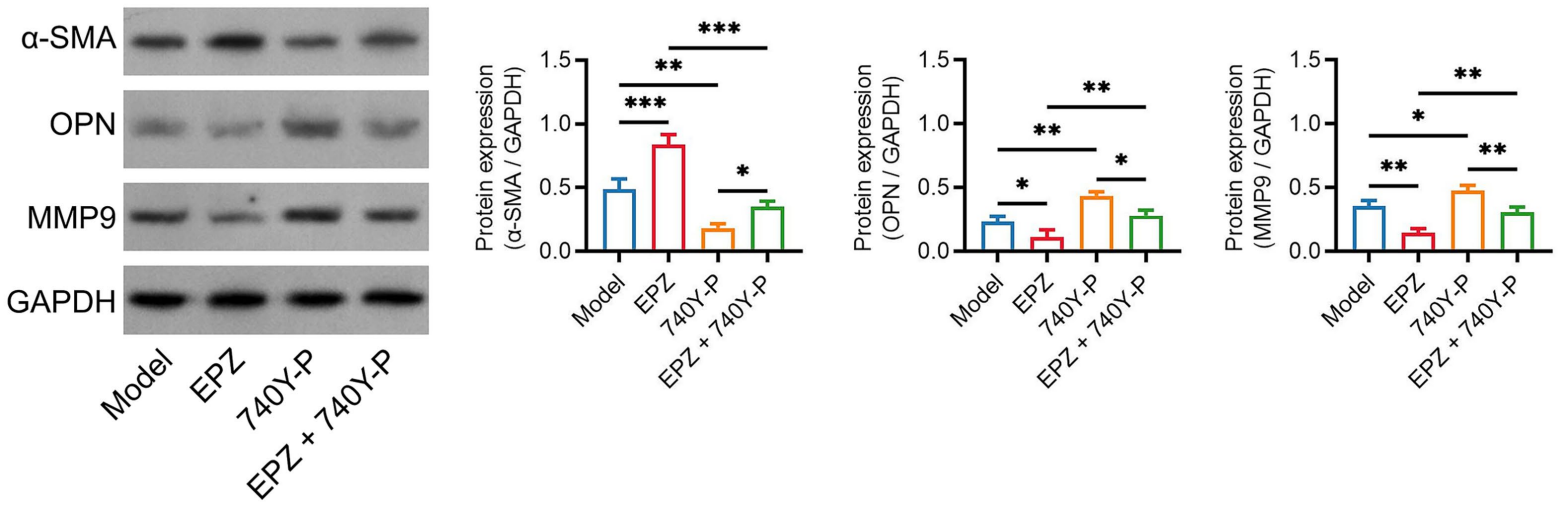
740Y-P reversed the effect of EPZ015666 on phenotype switching in Ang II-treated VSMCs. Comparison of α-SMA, OPN, and MMP9 levels among the model, EPZ, 740Y-P, and EPZ + 740Y-P groups. *: *p* < 0.05; **: *p* < 0.01; ***: *p* < 0.001.

Regarding phenotype markers, *α*-SMA protein level was decreased in the 740Y-P group vs. the Model group (*p* < 0.01), and in the EPZ + 740Y-P group vs. the EPZ group (*p* < 0.001). OPN and MMP9 protein levels were increased in the 740Y-P group vs. the Model group (both *p* < 0.05), and in the EPZ + 740Y-P group vs. the EPZ group (both *p* < 0.01) (Figure 5). The above findings indicated that 740Y-P promoted VSMC transition from the contractile phenotype to the synthetic phenotype. In addition, 740Y-P partially reversed the effect of EPZ015666 on VSMC phenotype switching.

**Figure 6:**
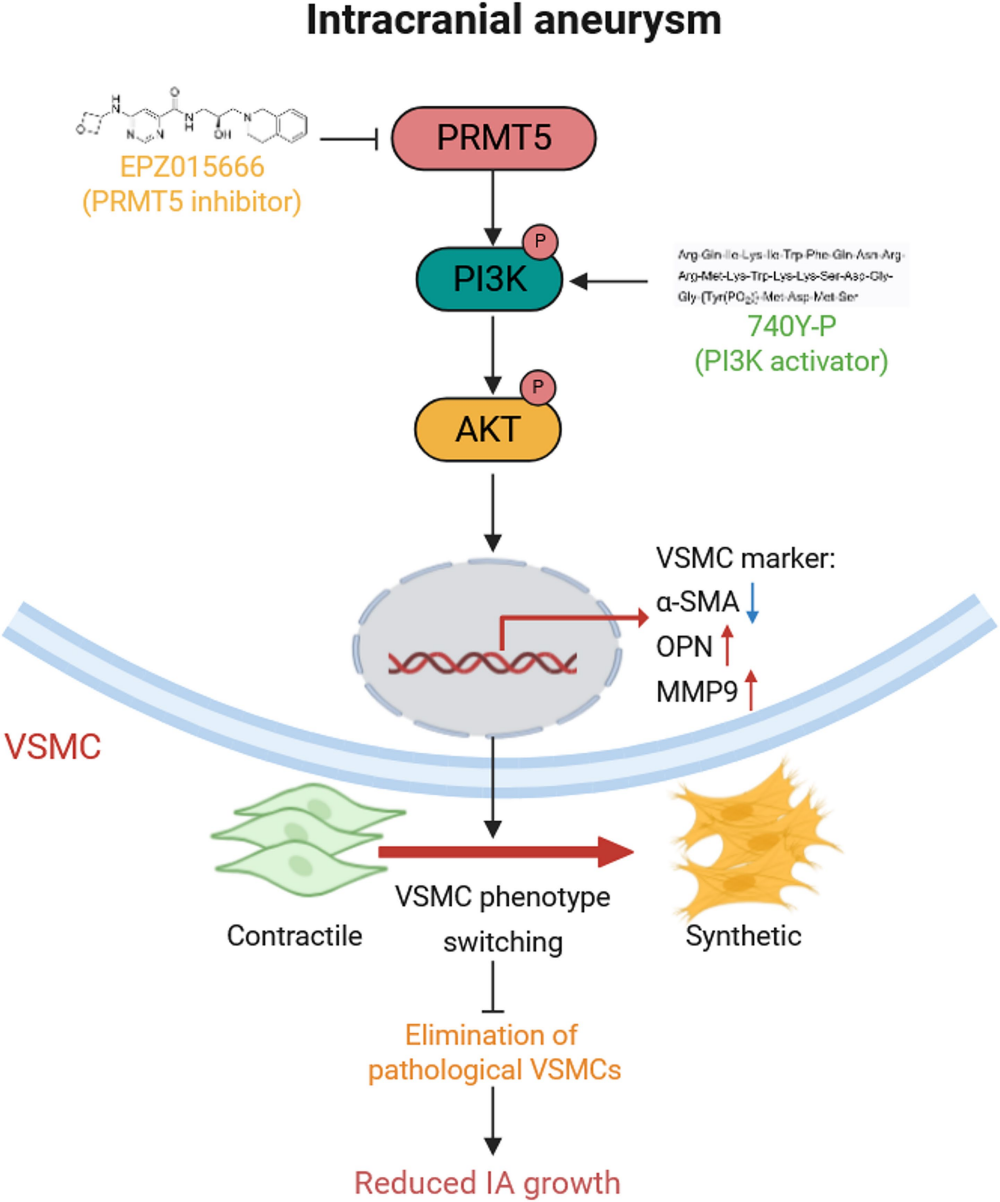
Schematic mechanism of EPZ015666 in IA pathology.

The schematic diagram exhibited that inhibition of PRMT5 by EPZ015666 suppressed the PI3K/AKT pathway to promote VSMC transition from the synthetic phenotype to the contractile phenotype (Figure 6).

## Discussion

The main findings of this study were as follows: (1) PRMT5 levels were increased in the IA rat and cell models; (2) EPZ015666 suppressed PRMT5 enzymatic activity, increased apoptosis, decreased viability and invasive capacity, facilitated the transition from the synthetic phenotype to the contractile phenotype, and inhibited the PI3K/AKT pathway in Ang II-treated VSMCs; (3) 740Y-P treatment reversed the effect of EPZ015666 on the above-mentioned processes in Ang II-treated VSMCs.

Previous studies have shown that PRMT5 is upregulated in several cardiovascular diseases (16, 20, 28). For example, a previous study found that PRMT5 was upregulated in human carotid aorta with stenosis and VSMCs of stenotic carotid arteries (28). Additionally, a highly expressed PRMT5 was observed in oxidized low-density lipoprotein (ox-LDL)-induced human umbilical vein endothelial cells (16). Furthermore, PRMT5 was overexpressed in human aortic SMCs, platelet-derived growth factor (PDGF)-stimulated VSMCs, human atherosclerotic lesions, and injured rat carotid arteries (20). However, the level of PRMT5 in IA was unknown. In this study, we initially established an IA rat model and discovered that PRMT5 was higher in the IA specimens of Model group than in the normal brain artery specimens of the Sham group. Additionally, IA cell model was constructed by treating rat brain artery VSMCs with Ang II. Then, we observed that the PRMT5 level was increased in the IA cell model groups compared to the Untreated group. *In vivo* and *in vitro* experiments showed that PRMT5 had a high level in IA, suggesting its potential involvement in IA progression.

EPZ015666, a small-molecule inhibitor of PRMT5 enzymatic activity, acts by blocking PRMT5-mediated protein methylation, thereby inhibiting its downstream cellular functions (21). The protective role of EPZ015666 in cardiovascular diseases has been disclosed by some previous studies (19, 20, 22). In the present study, we found that EPZ015666 did not affect PRMT5 level but decreased H4R3me2s in Ang II-treated VSMCs. This finding was in line with a previous study, which reported that EPZ015666 inhibited the enzymatic activity of PRMT5 without impacting the level of PRMT5 (29). Regarding the effect of EPZ015666 on cellular behaviors, we found that EPZ015666 enhanced apoptosis and reduced viability and invasive capacity in Ang II-treated VSMCs. The above findings were partly supported by a previous study, which indicated that PRMT5 silencing attenuated VSMC proliferation and migration in the cardiovascular disease model (20). Moreover, we found that EPZ015666 promoted the VSMC transition from the synthetic phenotype to the contractile phenotype, as evidenced by increased *α*-SMA, decreased OPN, and reduced MMP9. According to a previous study, we speculated that EPZ015666 might inhibit PRMT5-mediated histone arginine methylation to modulate the transcriptional serum response factor/myocardin/p300 complex, thereby affecting VSMC phenotypic markers (20).

The PI3K/AKT pathway has been identified as an important pathway in the formation of IA (30). As reported by a previous study, the inactivation of the PI3K/AKT pathway could regulate VSMC proliferation, apoptosis, and vascular remodeling to attenuate IA progression (31). Importantly, evidence suggested that PRMT5 could activate the PI3K/AKT pathway by mediating methylation of the p55 subunit of PI3K (32). However, whether the regulation of PRMT5 on the PI3K/AKT pathway contributed to IA progression was unknown. In this study, we found that the PI3K/AKT pathway was inhibited by EPZ015666 in Ang II-treated VSMCs. Then, we added 740Y-P, a PI3K/AKT pathway agonist, to EPZ015666 in Ang II-treated VSMCs. It was discovered that 740Y-P reversed the effect of EPZ015666 on VSMC behaviors and phenotype switching, which suggested that EPZ015666 inhibited the PI3K/AKT pathway to regulate these processes, thereby retarding IA progression. Of note, the reversal effects of 740Y-P on EPZ015666 were partial, indicating that additional signaling pathways beyond PI3K/AKT may also contribute to the regulatory effects of EPZ015666 on VSMC behavior and phenotype. Although the present study did not directly investigate the molecular mechanism by which PRMT5 regulated the PI3K/AKT pathway, previous evidence suggested that PRMT5 might modulate PI3K activity by influencing the methylation of the PI3K p55 subunit (33). Additionally, PRMT5 might also methylate KLF4, thereby inhibiting its ubiquitination and enhancing its protein stability, which would further influence downstream signaling pathways, such as PI3K/AKT pathway (34). However, these potential mechanisms require further experimental validation.

Currently, pharmacokinetic data regarding the blood–brain barrier (BBB) penetration of EPZ015666 are not available. Although EPZ015666 has shown *in vivo* efficacy in various disease models (19, 20), its central nervous system exposure and distribution in cerebrovascular tissues remain unclear. Therefore, the extent to which EPZ015666 directly penetrates the BBB and exerts effects within intracranial vascular lesions warrants further investigation. Future studies incorporating quantitative pharmacokinetic analyses will help clarify this issue and strengthen the interpretation of *in vivo* efficacy in IA models. Moreover, as PRMT5 shares structural similarities with other PRMTs, potential off-target effects of EPZ015666 on PRMT1 or PRMT3 cannot be completely excluded. Such non-selective inhibition may contribute to hematological toxicities, which warrants careful evaluation in future studies. To enhance therapeutic specificity and reduce systemic exposure, localized or targeted delivery strategies, such as nanoparticle-based delivery systems, may be considered (35).

Several limitations existed in this study. (1) Only male rats were used in the IA model, which limited the generalizability of our findings, particularly with respect to potential sex-specific differences. (2) The follow-up period was limited (< 8 weeks), which might not fully reflect long-term IA progression. (3) Aneurysm rupture was not assessed as an experimental end-point. (4) No priori power calculation was performed, and the sample size (*n* = 5 per group) might limit the statistical power. (5) Only a limited range of EPZ015666 concentrations (0–10 μM) was evaluated in this study, which restricted us to fully investigate its pharmacological effects across different concentrations on VSMCs.

## Conclusion

In conclusion, EPZ015666 suppresses IA progression by promoting VSMC apoptosis, reducing viability and invasive capacity, and facilitating the transition from synthetic phenotype to contractile phenotype via inhibiting the PI3K/AKT pathway. Our findings offer insights that EPZ015666 may serve as a novel pharmacologic therapy in patients with IA.

## Data Availability

The original contributions presented in the study are included in the article/Supplementary material, further inquiries can be directed to the corresponding author.

## Supplementary material

SUPPLEMENTARY FIGURE 1 Confirmation of IA formation. Representative images of cerebral arteries visualized by bromophenol blue-gelatin perfusion and comparison of IA incidence between the Sham and Model groups. *: *p* < 0.05.

SUPPLEMENTARY FIGURE 2 PI3K level was increased in IA models. *In vivo* analysis of p-PI3K level between the Sham and Model groups **(A)**. *In vitro* analysis of p-PI3K level among the Untreated, Ang II (0.1 μM), Ang II (0.5 μM), and Ang II (1 μM) groups **(B)**. *: *p* < 0.05; **: *p* < 0.01; ns: not significant.

SUPPLEMENTARY FIGURE 3 Dose–response curve of EPZ015666 on H4R3me2s levels. EPZ-induced dose-dependent reduction in H4R3me2s levels (IC_50_ = 3.850 ± 0.785 μM).

SUPPLEMENTARY FIGURE 4 Confirmation of the effect of EPZ015666 on apoptosis and invasive capacity in Ang II-treated VSMCs. Comparison of cleaved caspase-3 among the Model, EPZ, 740Y-P, and EPZ + 740Y-P groups **(A)**. Comparison of invasive capacity using a shortened incubation time (6 h), distinct from the primary 24 h Transwell assay, among the model, EPZ, 740Y-P, and EPZ + 740Y-P groups **(B)**. *: *p* < 0.05; **: *p* < 0.01.
