# Supplementary figures and images for: PRMT5 inhibition suppresses the PI3K/AKT pathway to attenuate vascular smooth muscle cell pathological phenotype in intracranial aneurysm
Source: Front Neurol. 2026 Jun 18;17:1771196. doi: 10.3389/fneur.2026.1771196 (PMC13322850; doi:10.3389/fneur.2026.1771196)

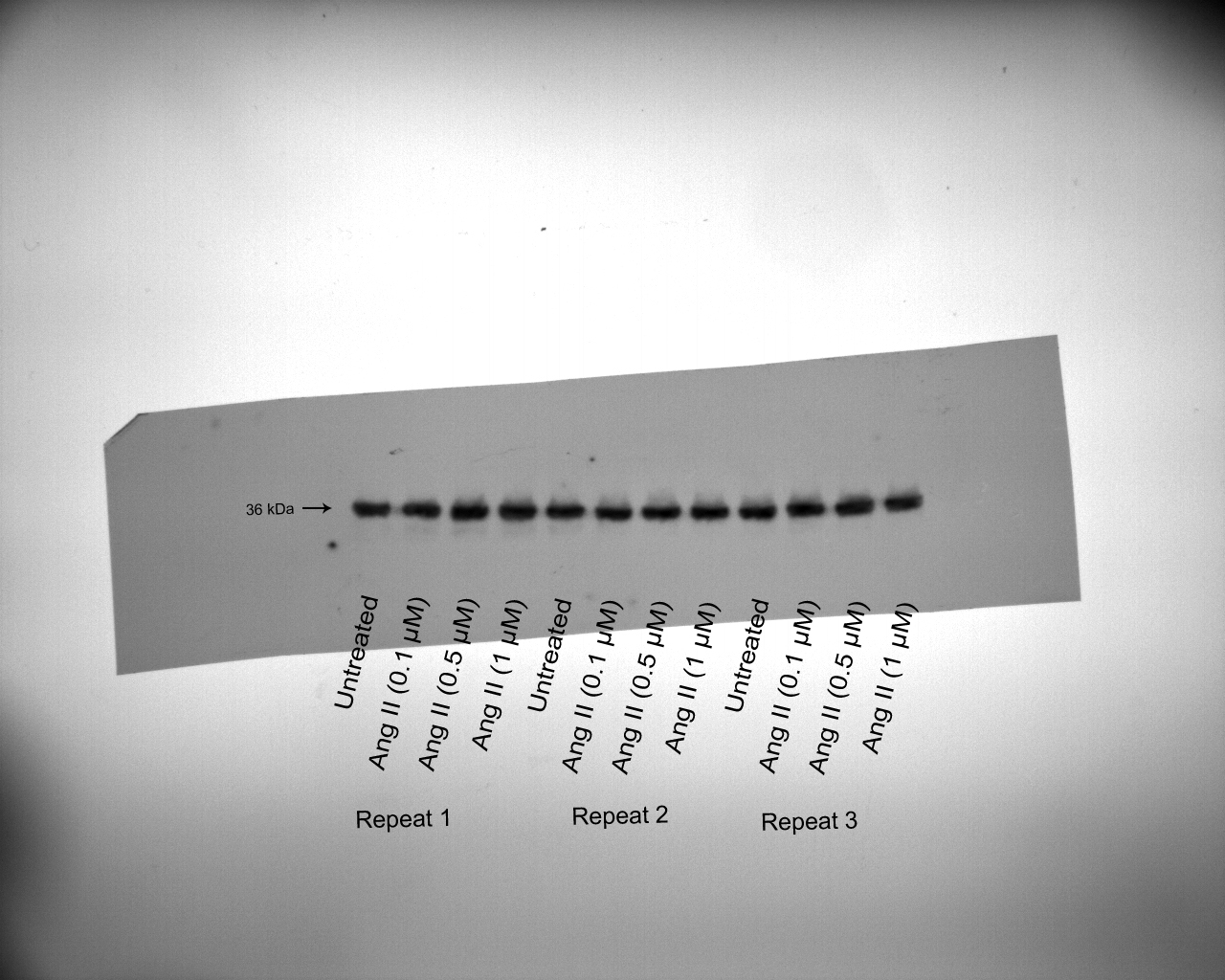

Supplement: Supplementary file 2 [file Data_Sheet_2.ZIP › Uncropped Western Blot Images/Fig1C_GAPDH.jpg]

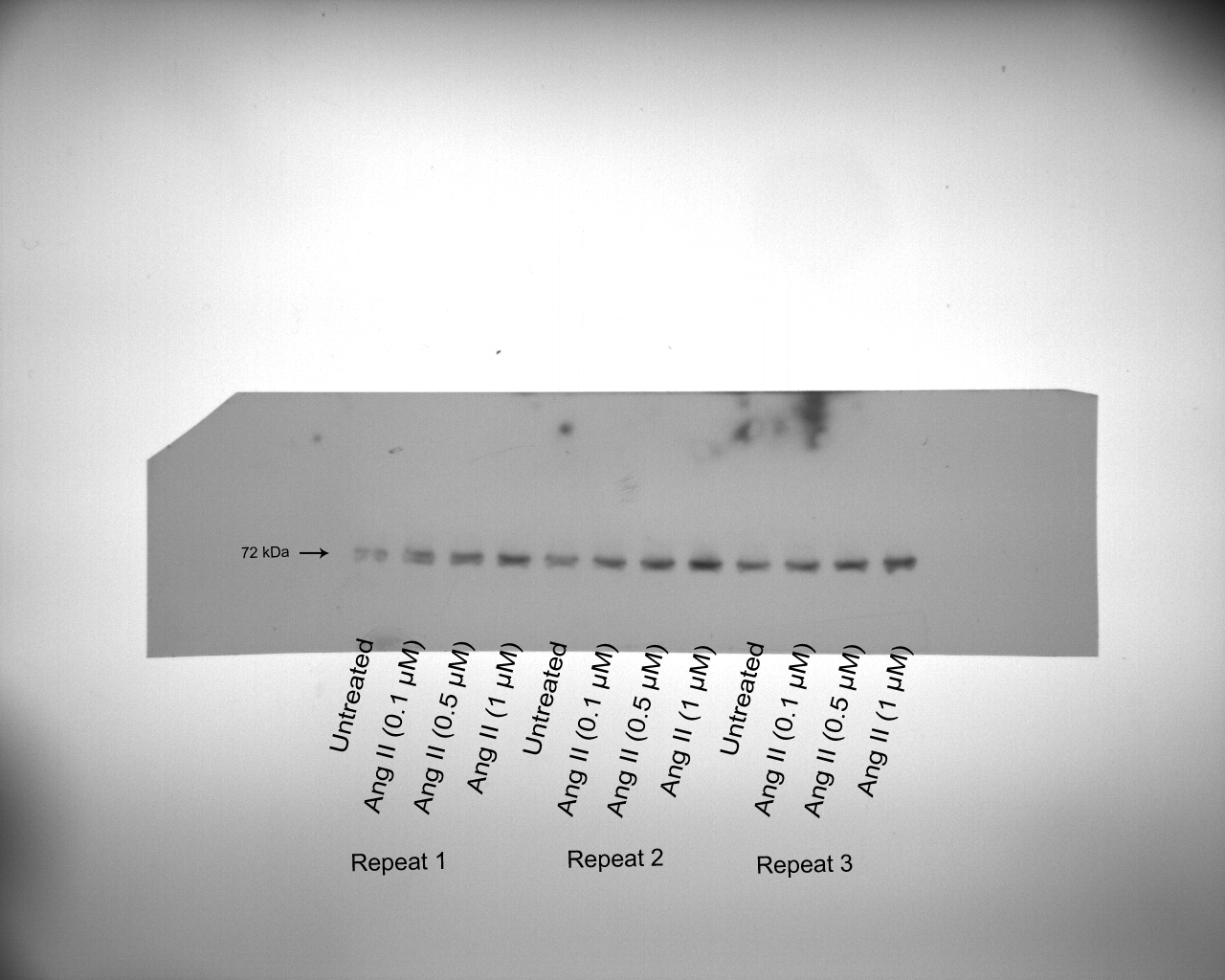

Supplement: Supplementary file 2 [file Data_Sheet_2.ZIP › Uncropped Western Blot Images/Fig1C_PRMT5.jpg]

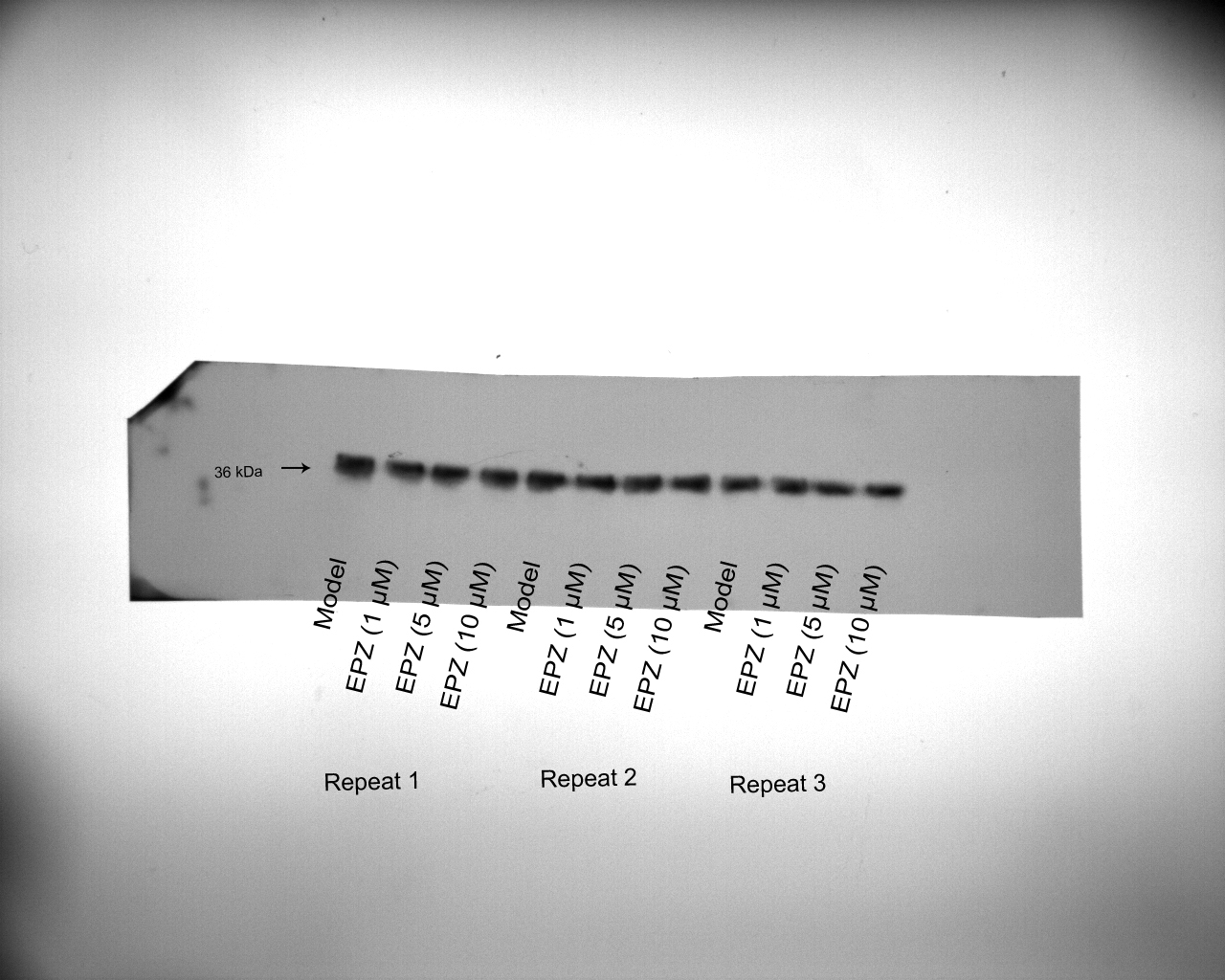

Supplement: Supplementary file 2 [file Data_Sheet_2.ZIP › Uncropped Western Blot Images/Fig3A_GAPDH.jpg]

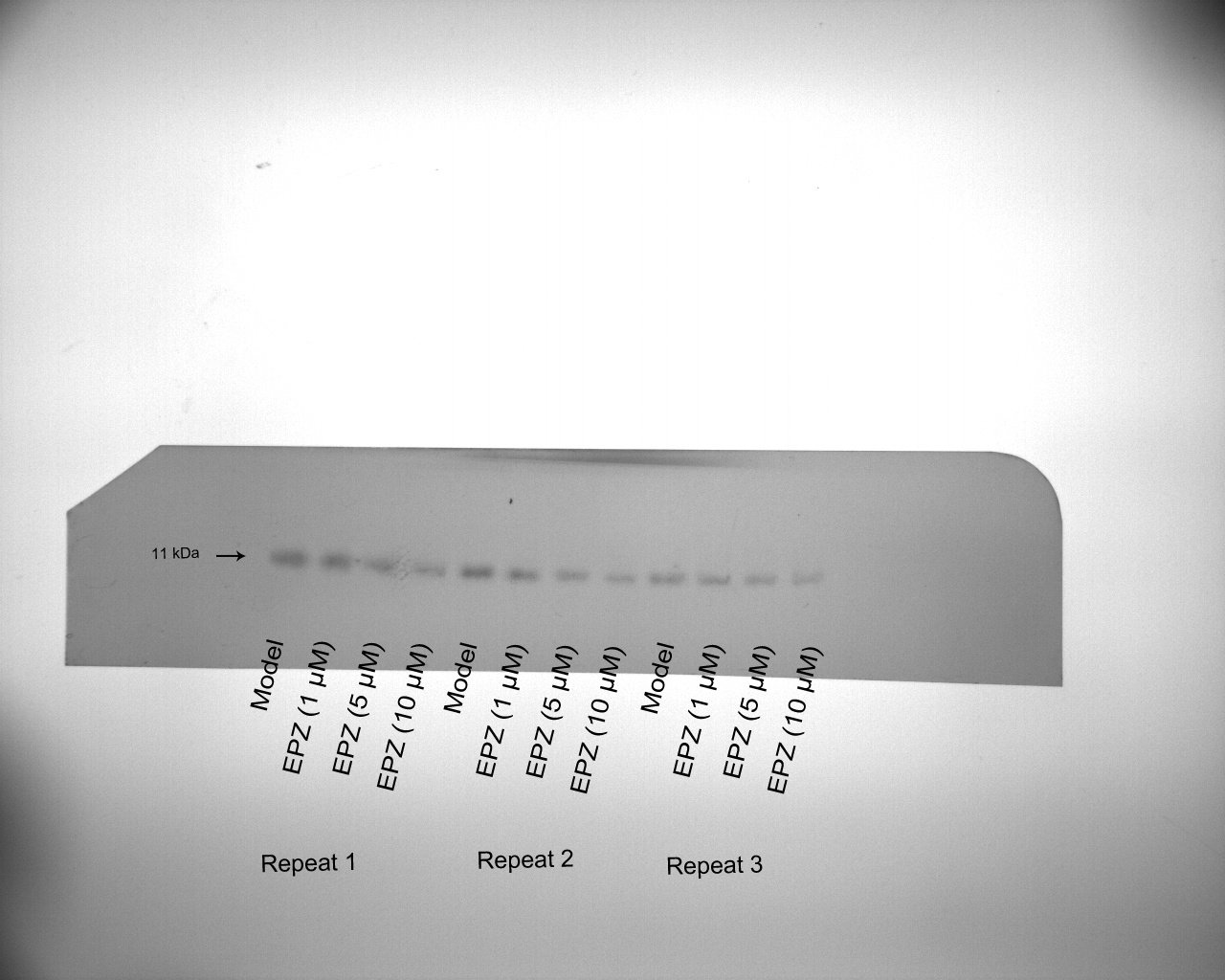

Supplement: Supplementary file 2 [file Data_Sheet_2.ZIP › Uncropped Western Blot Images/Fig3A_H4R3me2s.jpg]

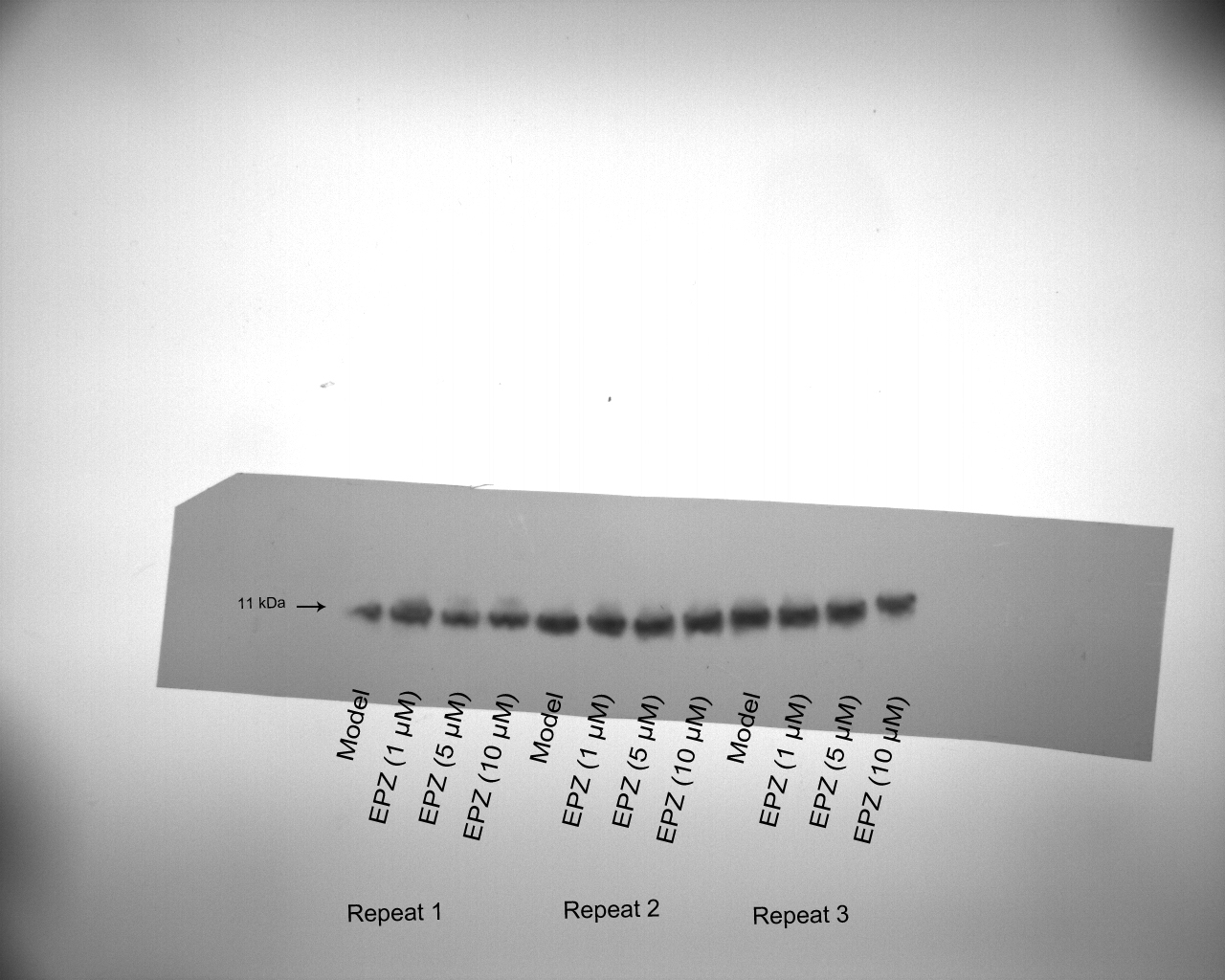

Supplement: Supplementary file 2 [file Data_Sheet_2.ZIP › Uncropped Western Blot Images/Fig3A_Histone H4.jpg]

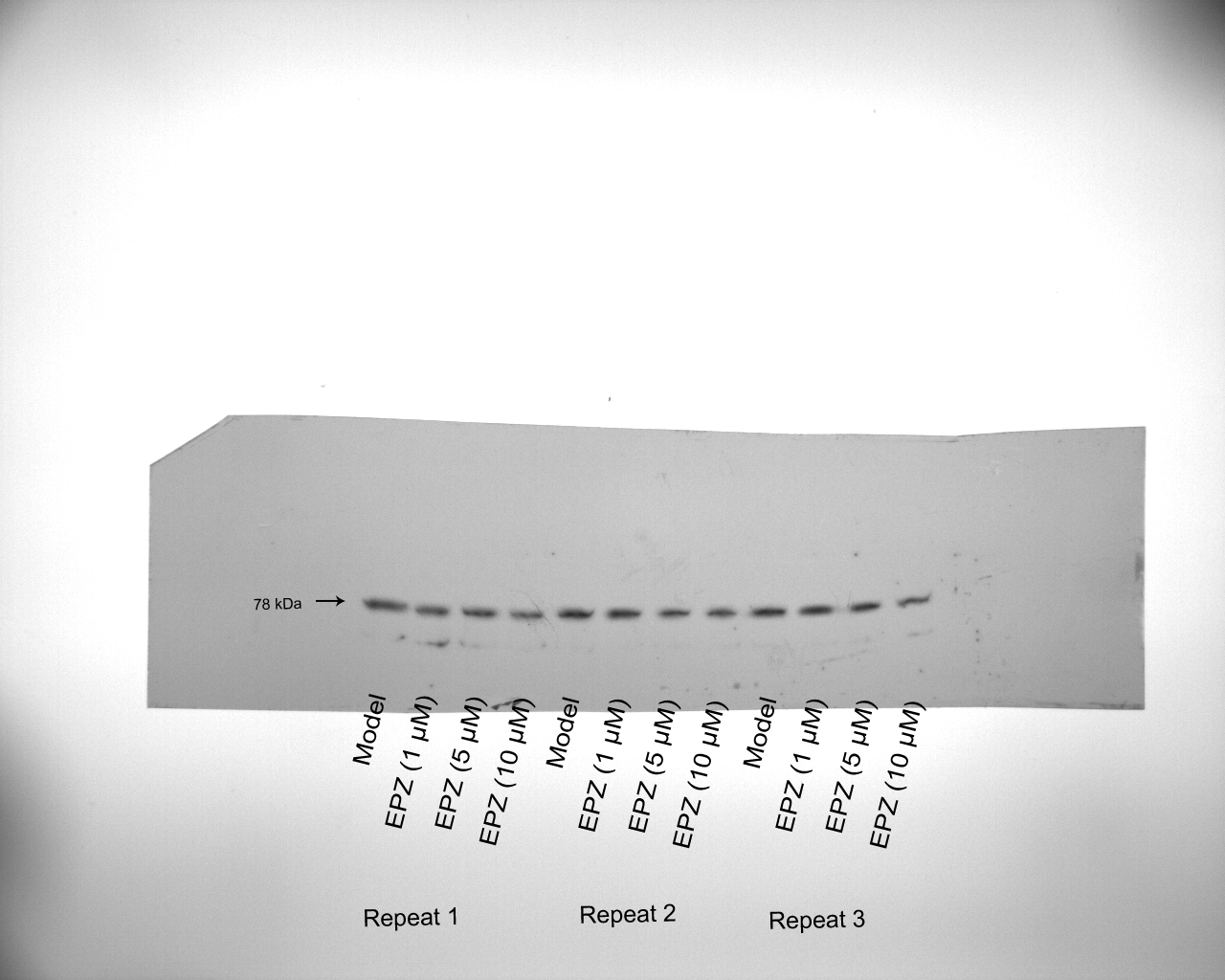

Supplement: Supplementary file 2 [file Data_Sheet_2.ZIP › Uncropped Western Blot Images/Fig3A_MMP9.jpg]

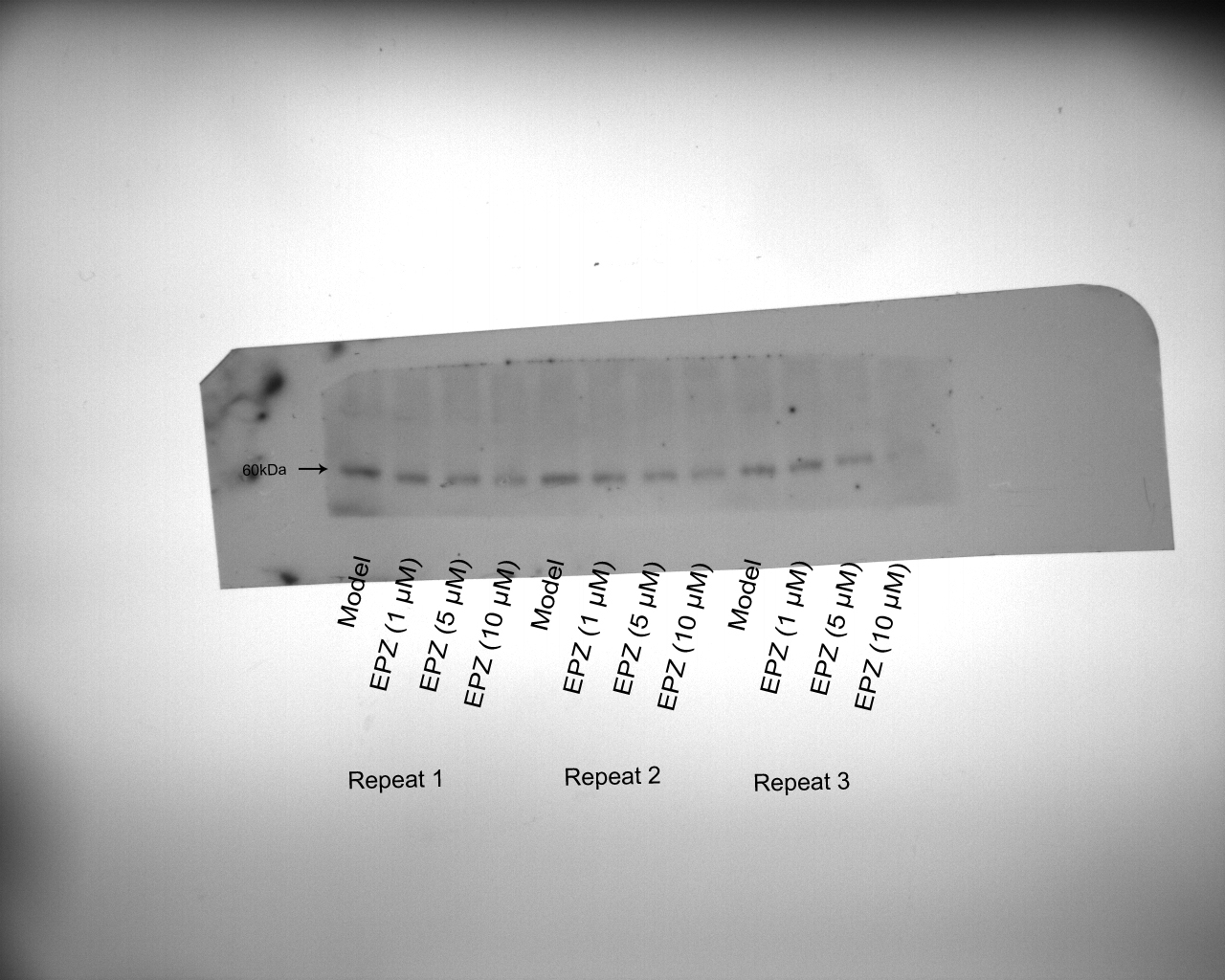

Supplement: Supplementary file 2 [file Data_Sheet_2.ZIP › Uncropped Western Blot Images/Fig3A_OPN.jpg]

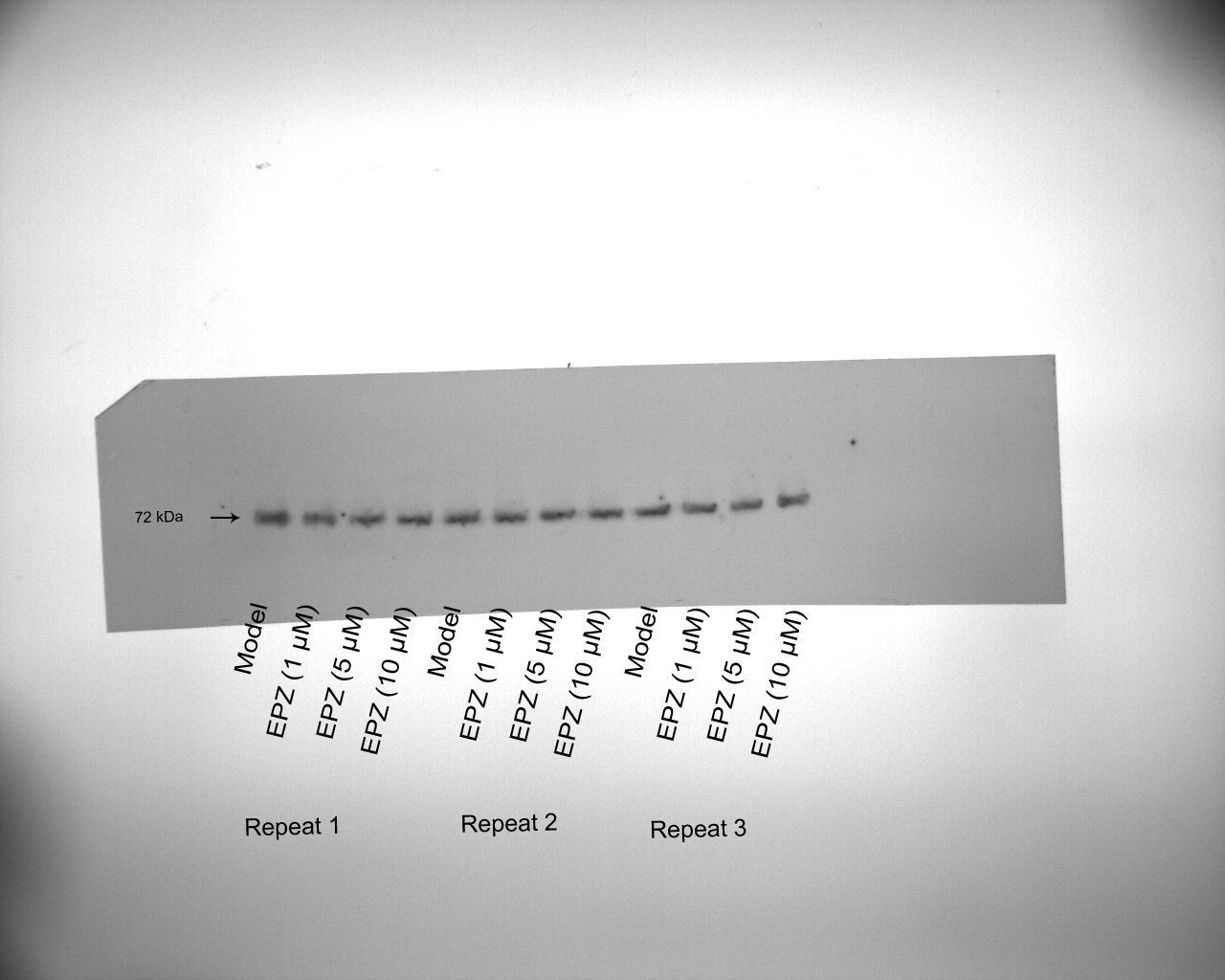

Supplement: Supplementary file 2 [file Data_Sheet_2.ZIP › Uncropped Western Blot Images/Fig3A_PRMT5.jpg]

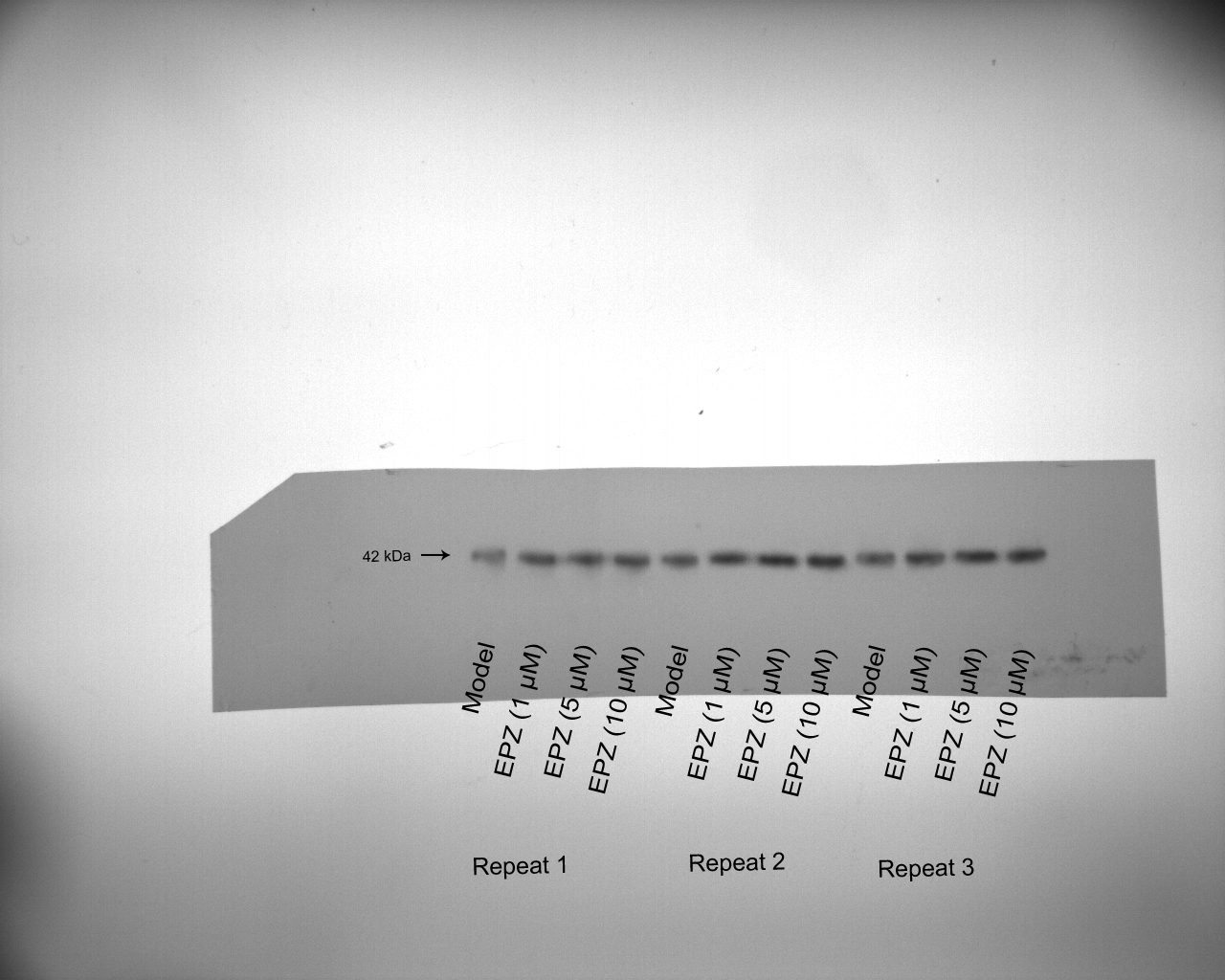

Supplement: Supplementary file 2 [file Data_Sheet_2.ZIP › Uncropped Western Blot Images/Fig3A_α-SMA.jpg]

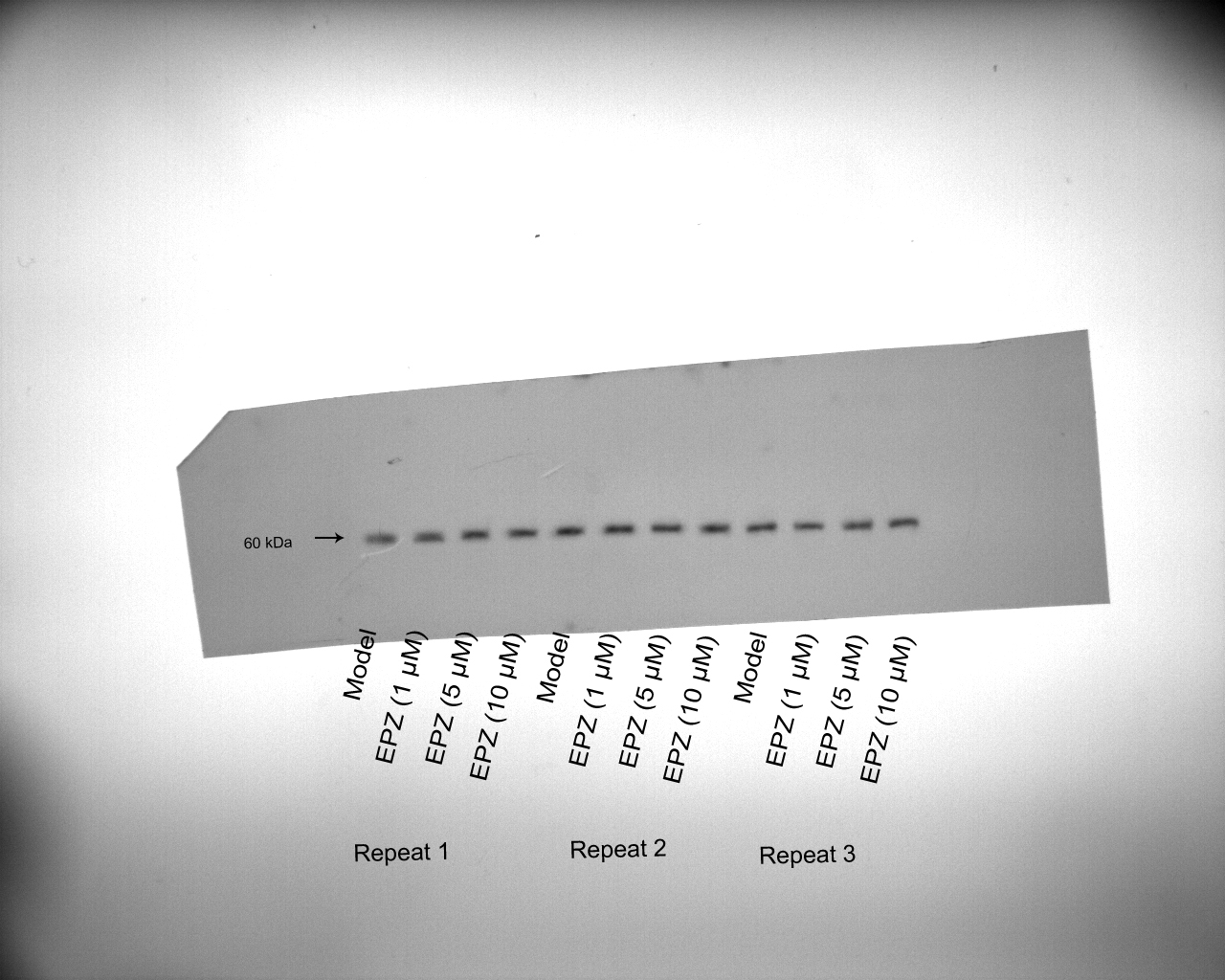

Supplement: Supplementary file 2 [file Data_Sheet_2.ZIP › Uncropped Western Blot Images/Fig3B_AKT.jpg]

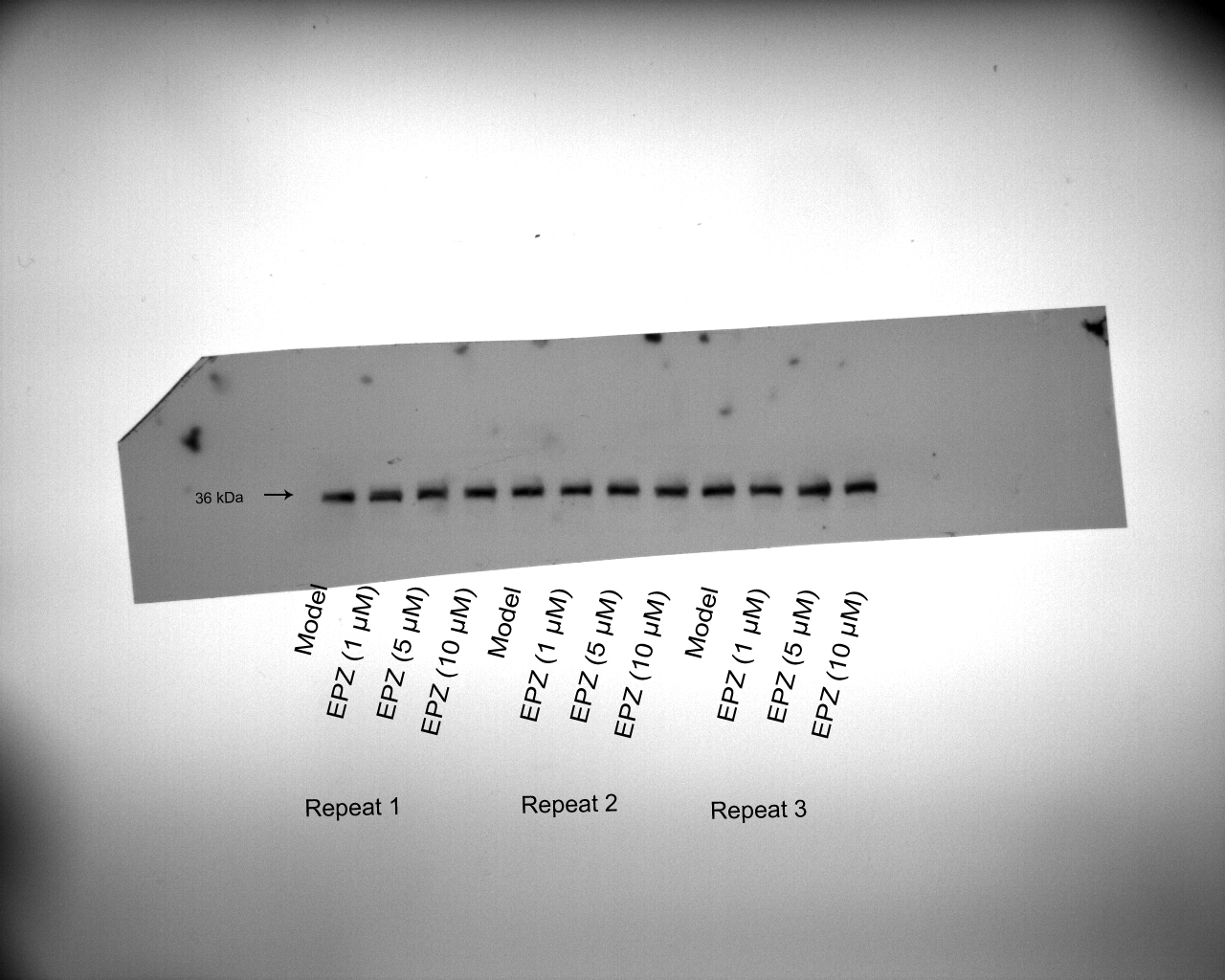

Supplement: Supplementary file 2 [file Data_Sheet_2.ZIP › Uncropped Western Blot Images/Fig3B_GAPDH.jpg]

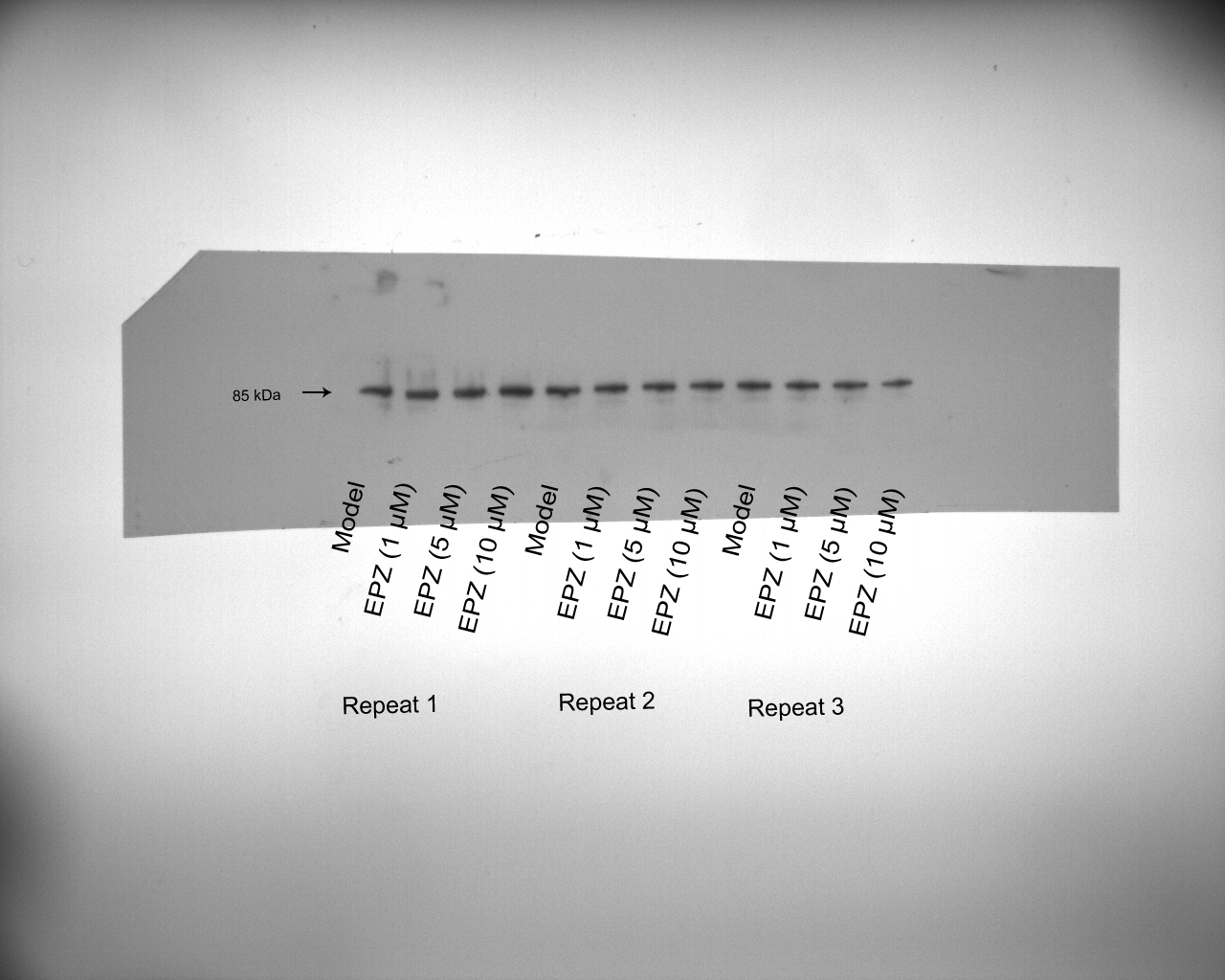

Supplement: Supplementary file 2 [file Data_Sheet_2.ZIP › Uncropped Western Blot Images/Fig3B_PI3K.jpg]

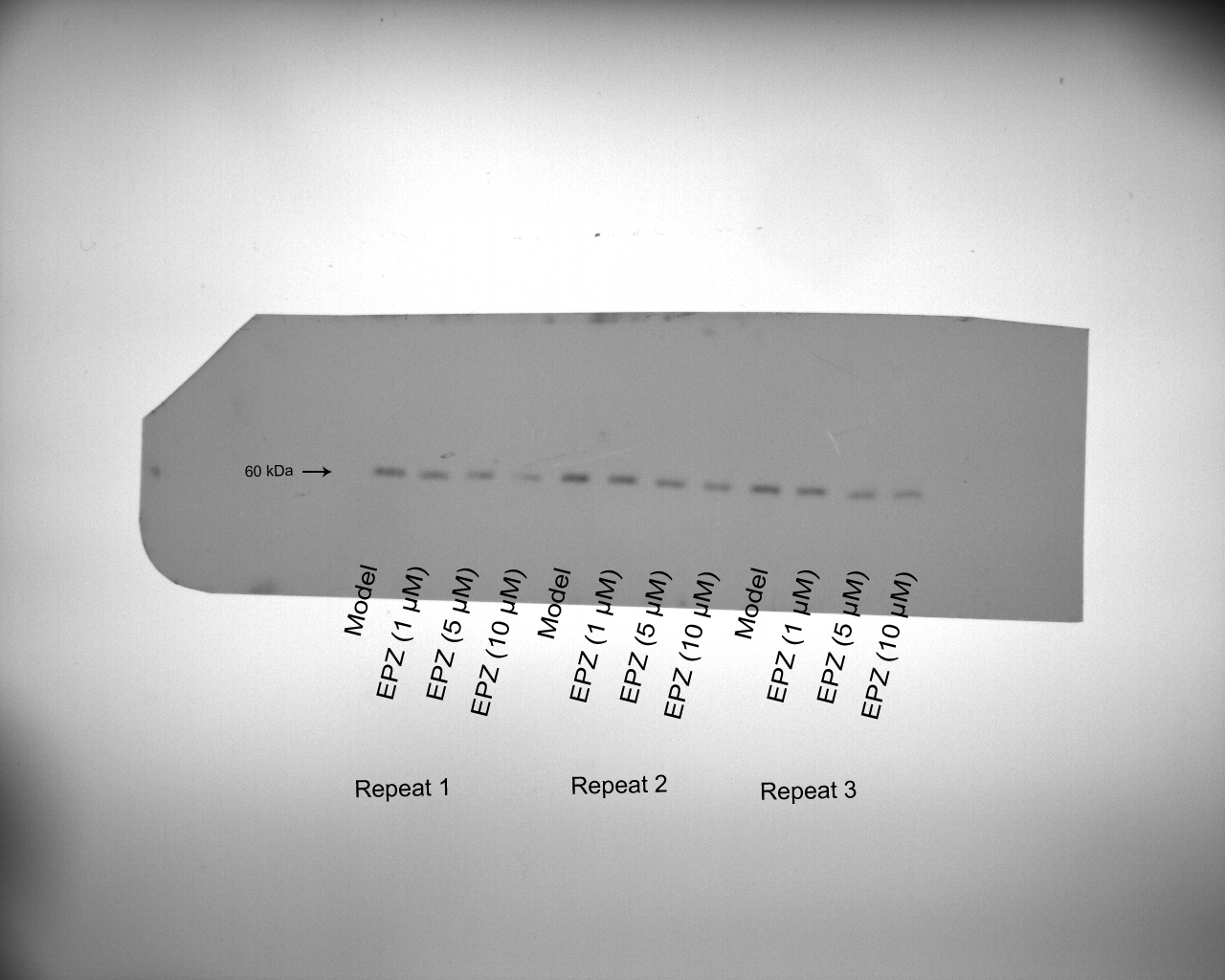

Supplement: Supplementary file 2 [file Data_Sheet_2.ZIP › Uncropped Western Blot Images/Fig3B_p-AKT.jpg]

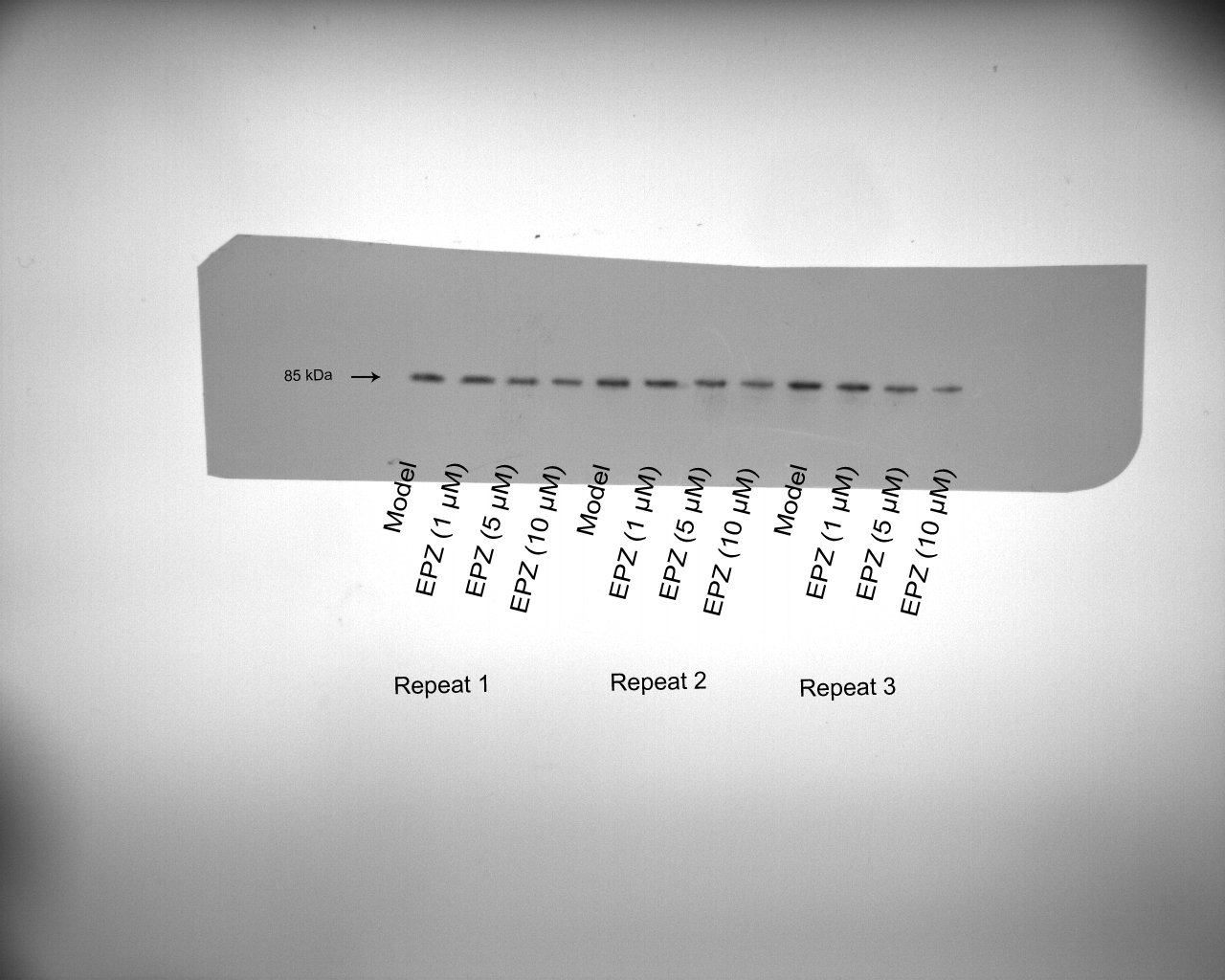

Supplement: Supplementary file 2 [file Data_Sheet_2.ZIP › Uncropped Western Blot Images/Fig3B_p-PI3K.jpg]

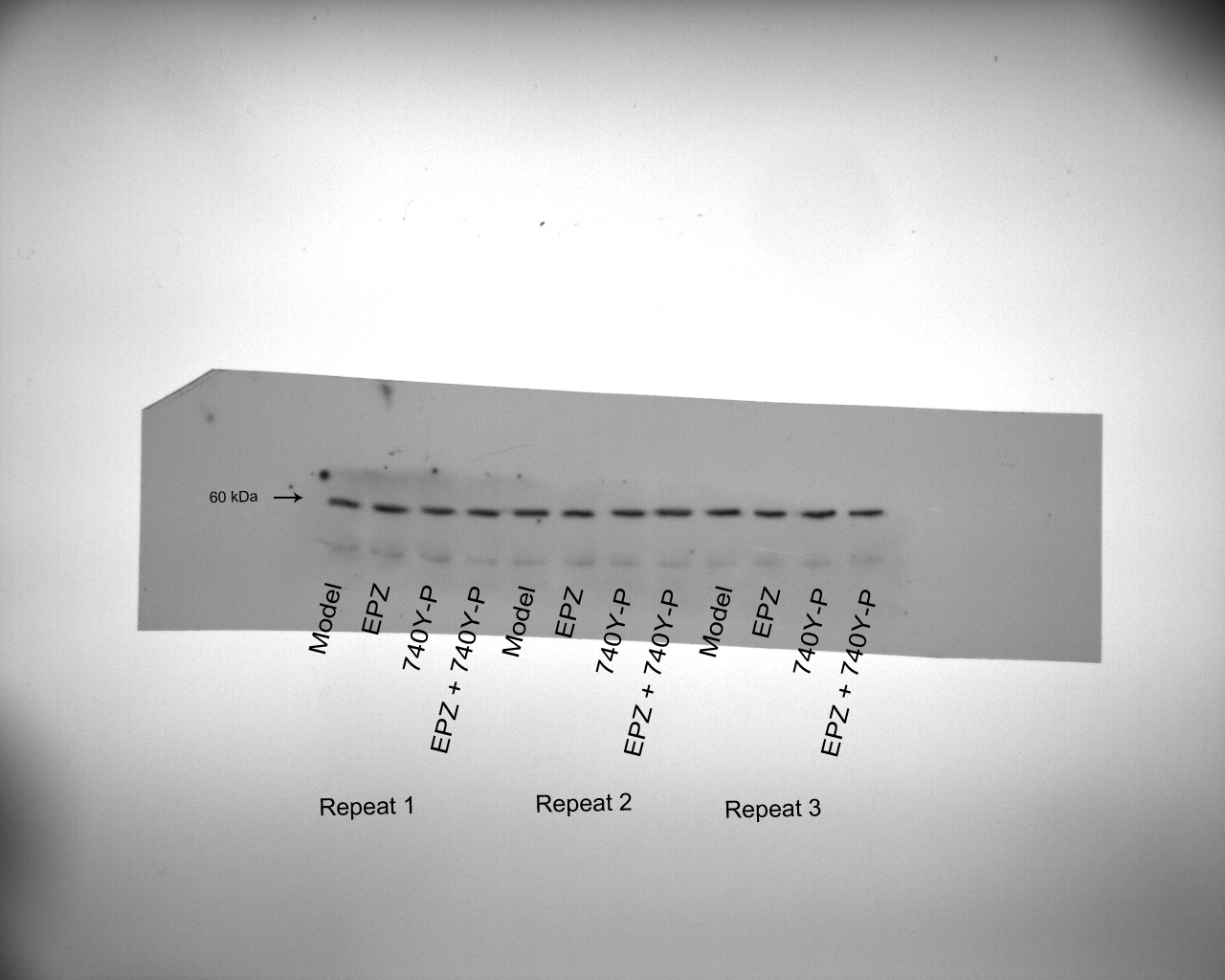

Supplement: Supplementary file 2 [file Data_Sheet_2.ZIP › Uncropped Western Blot Images/Fig4A_AKT.jpg]

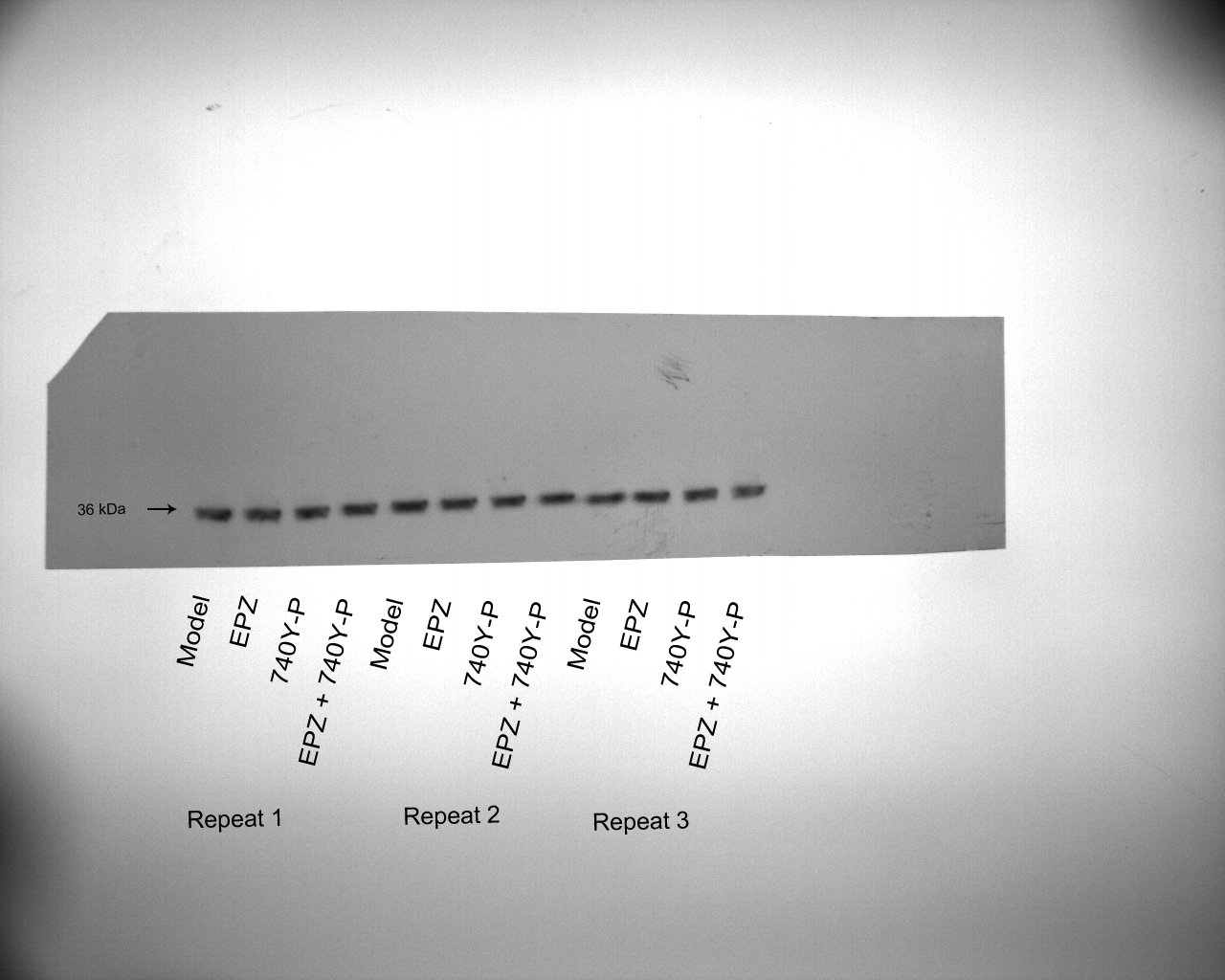

Supplement: Supplementary file 2 [file Data_Sheet_2.ZIP › Uncropped Western Blot Images/Fig4A_GAPDH.jpg]

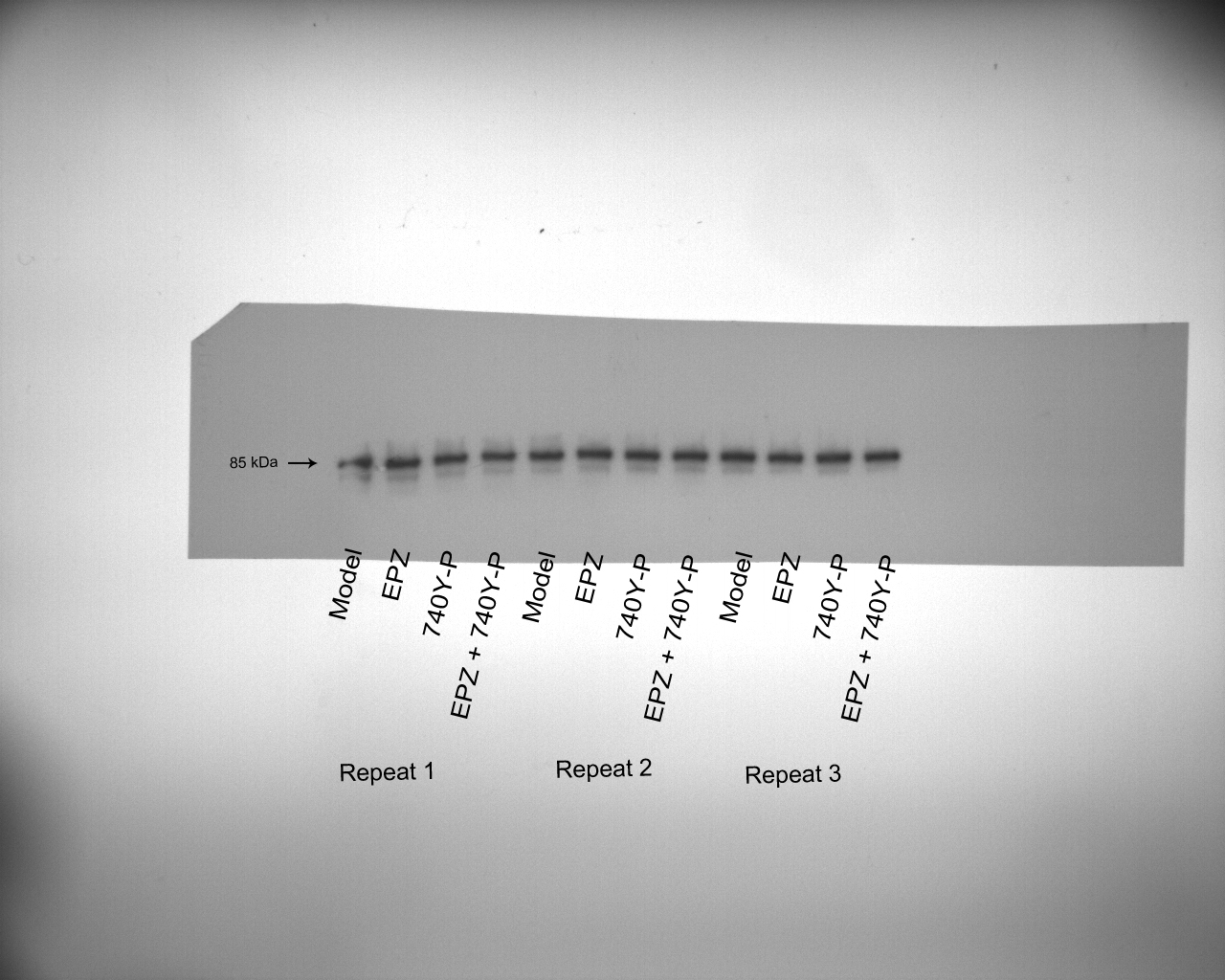

Supplement: Supplementary file 2 [file Data_Sheet_2.ZIP › Uncropped Western Blot Images/Fig4A_PI3K.jpg]

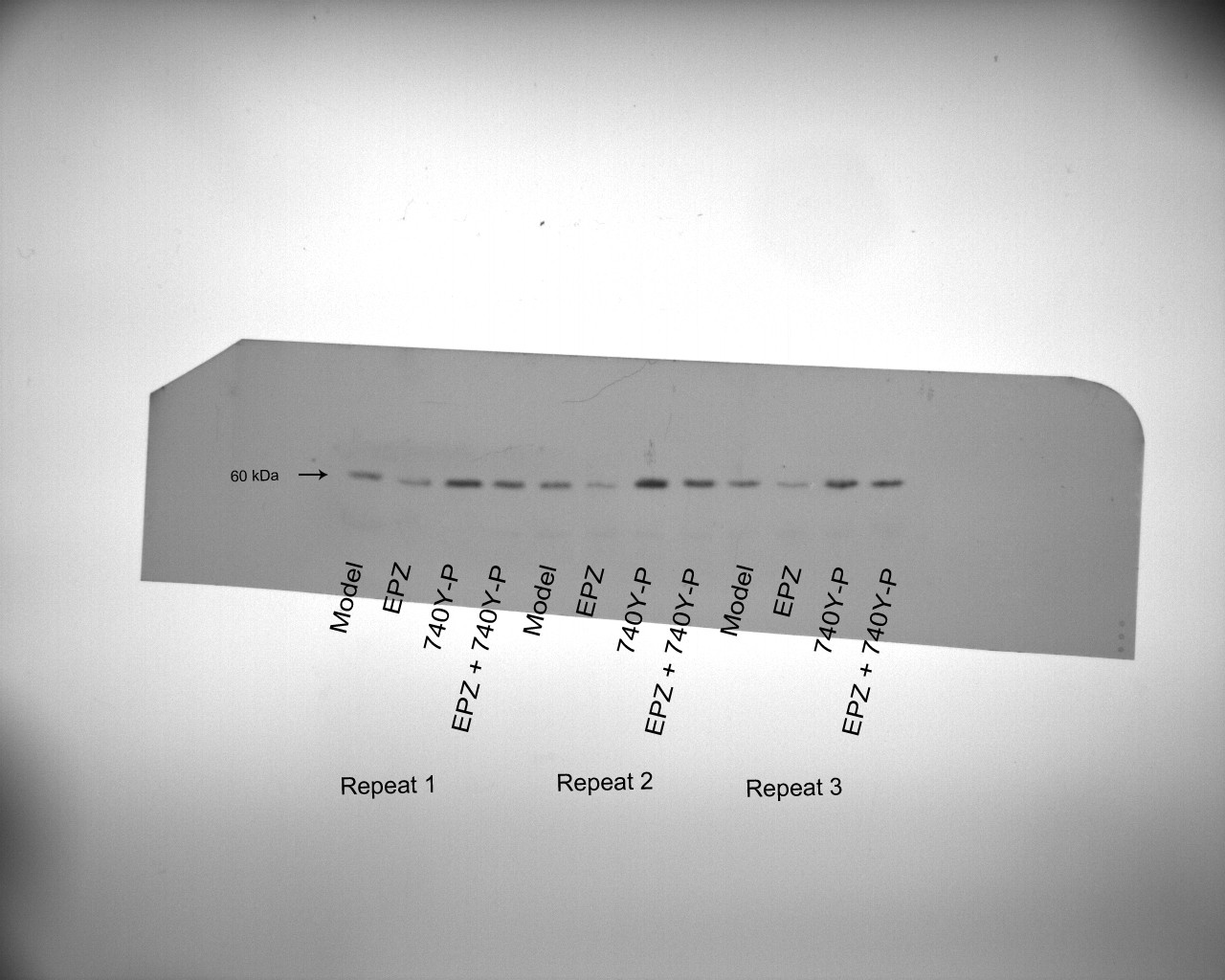

Supplement: Supplementary file 2 [file Data_Sheet_2.ZIP › Uncropped Western Blot Images/Fig4A_p-AKT.jpg]

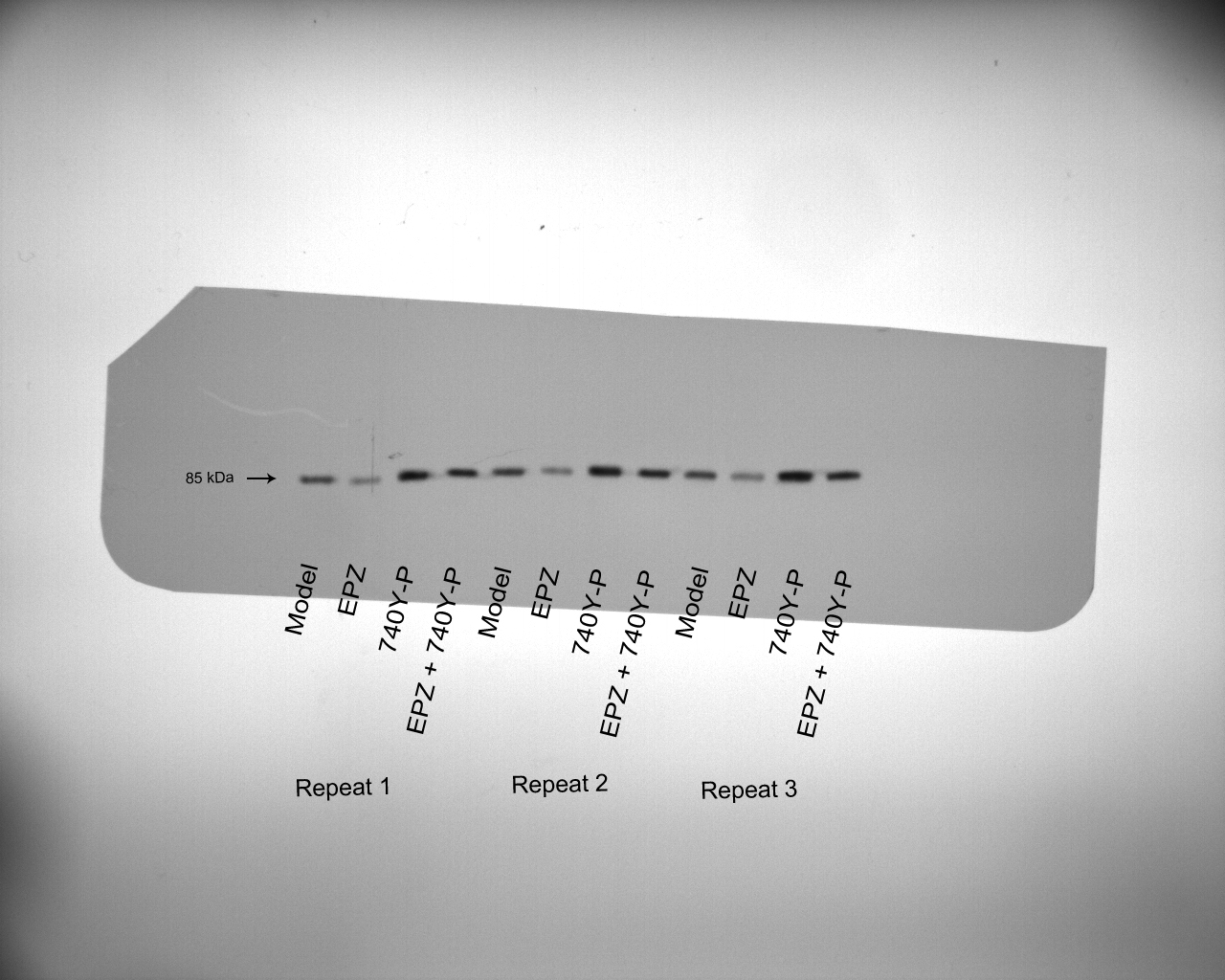

Supplement: Supplementary file 2 [file Data_Sheet_2.ZIP › Uncropped Western Blot Images/Fig4A_p-PI3K.jpg]

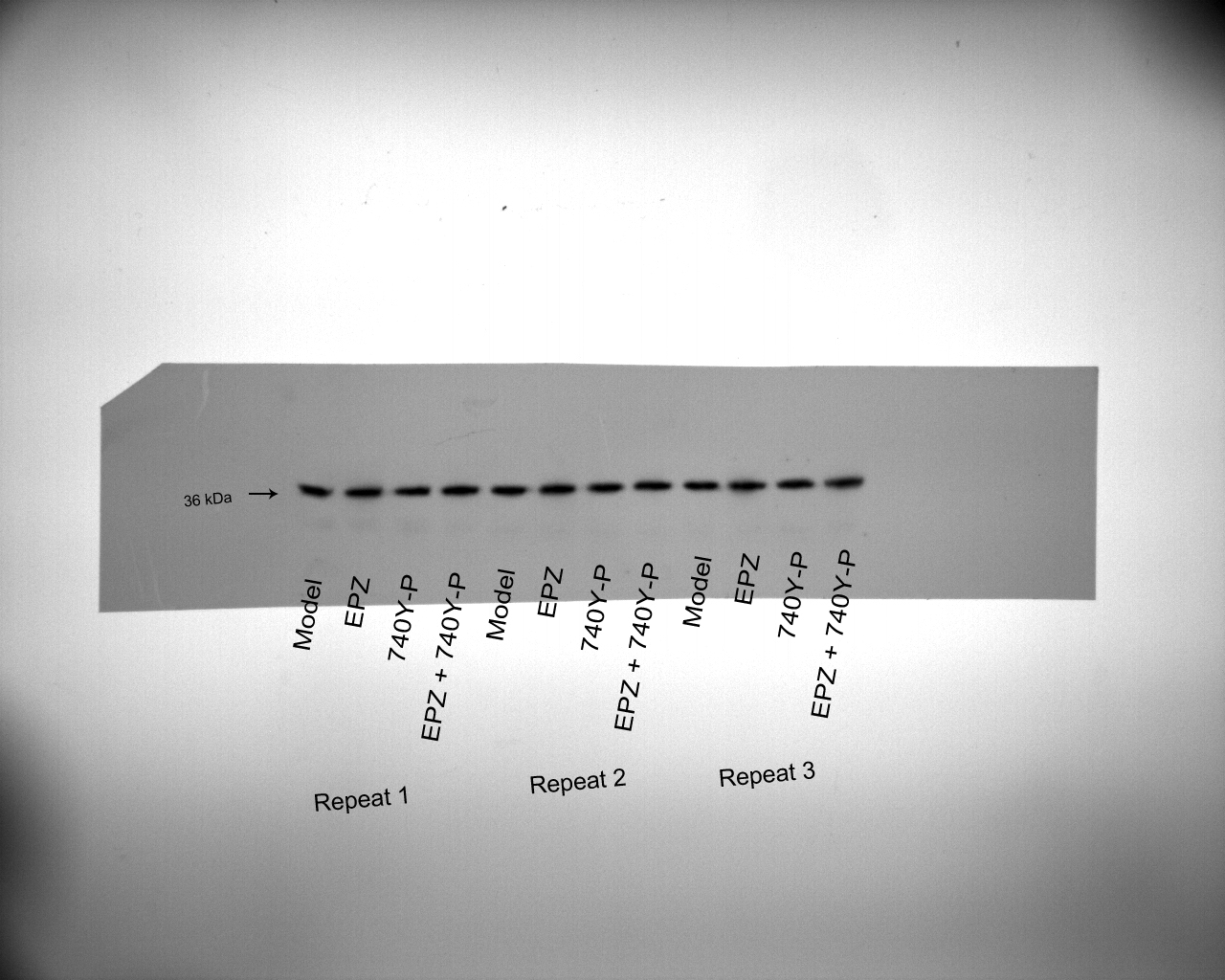

Supplement: Supplementary file 2 [file Data_Sheet_2.ZIP › Uncropped Western Blot Images/Fig5_GAPDH.jpg]

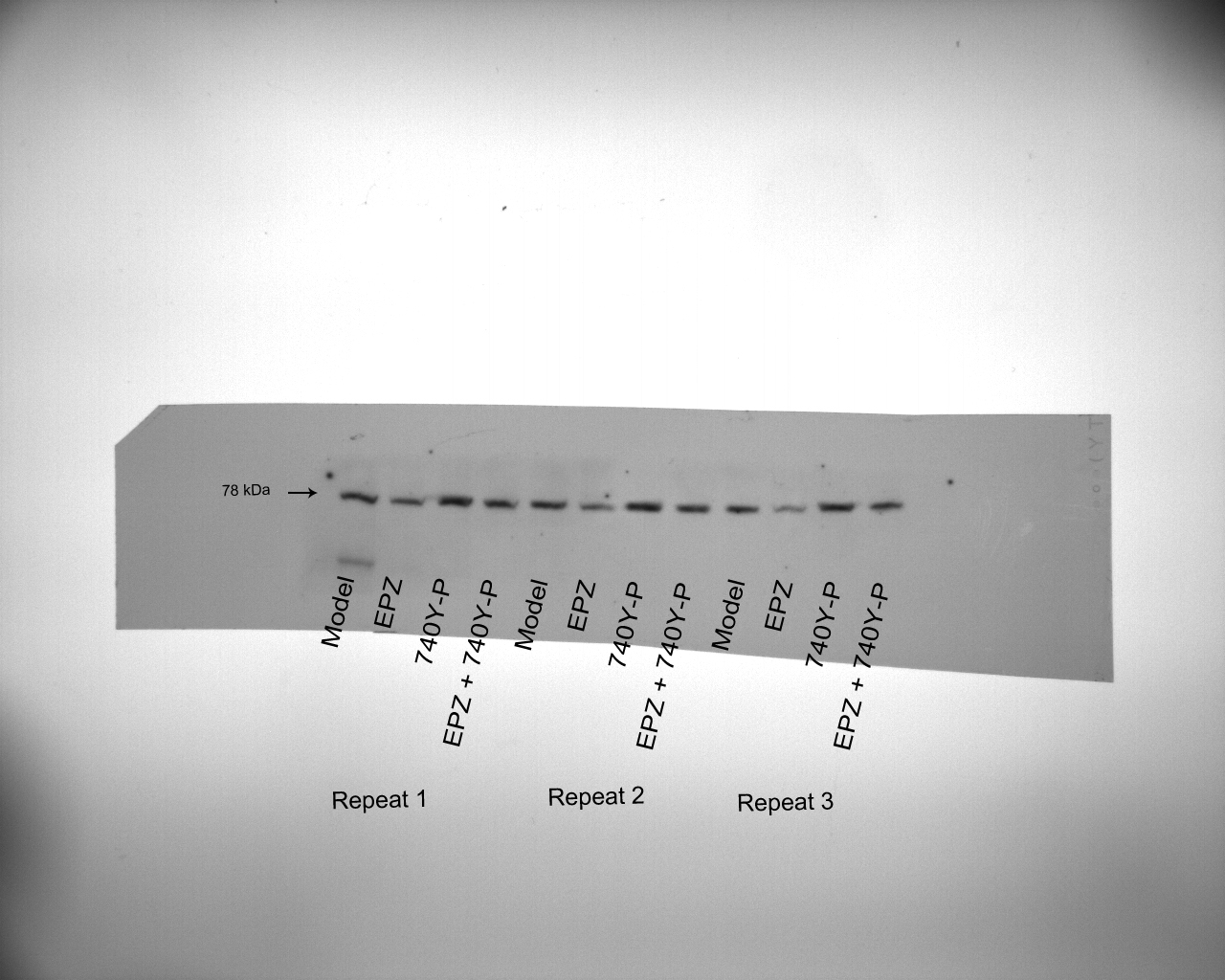

Supplement: Supplementary file 2 [file Data_Sheet_2.ZIP › Uncropped Western Blot Images/Fig5_MMP9.jpg]

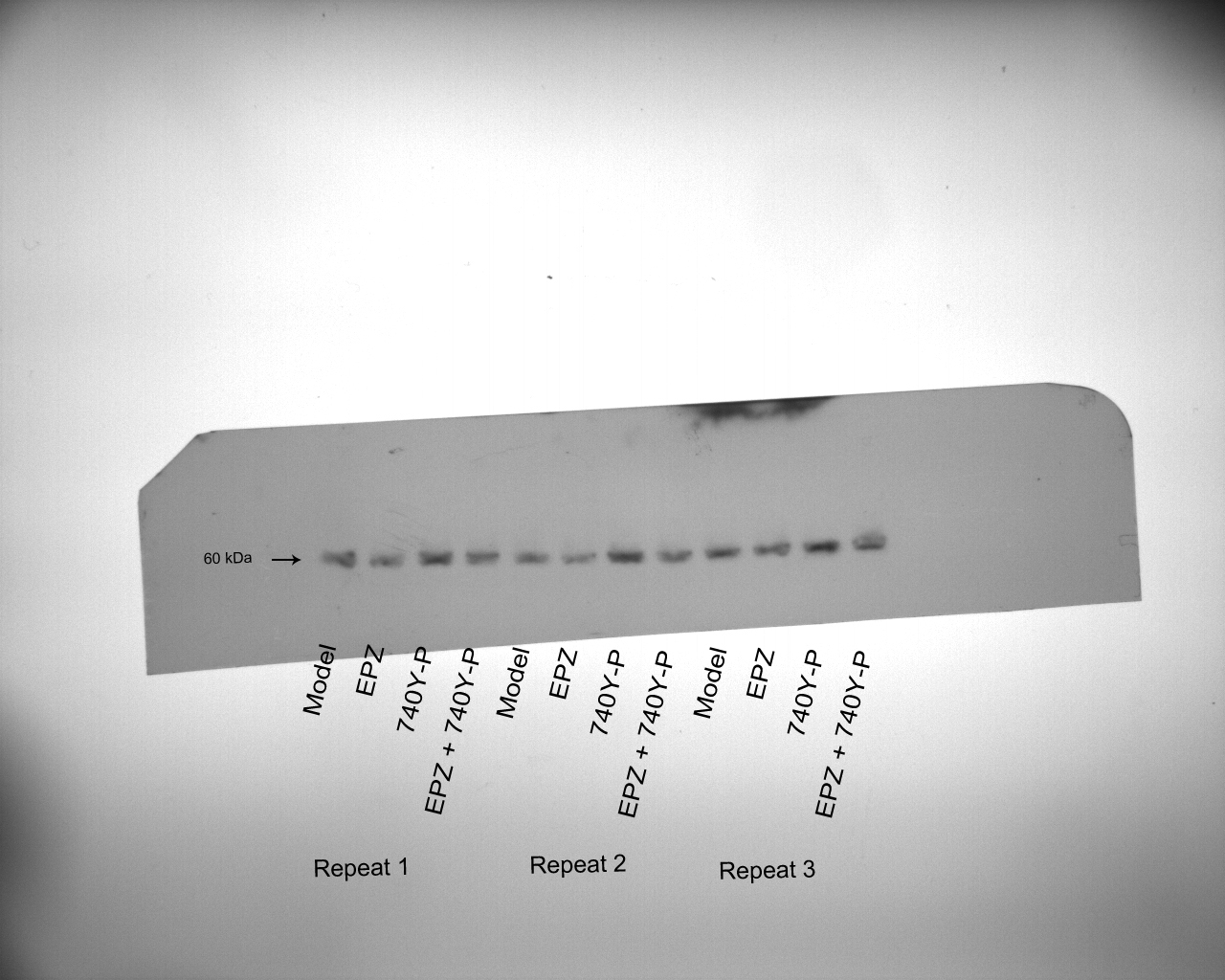

Supplement: Supplementary file 2 [file Data_Sheet_2.ZIP › Uncropped Western Blot Images/Fig5_OPN.jpg]

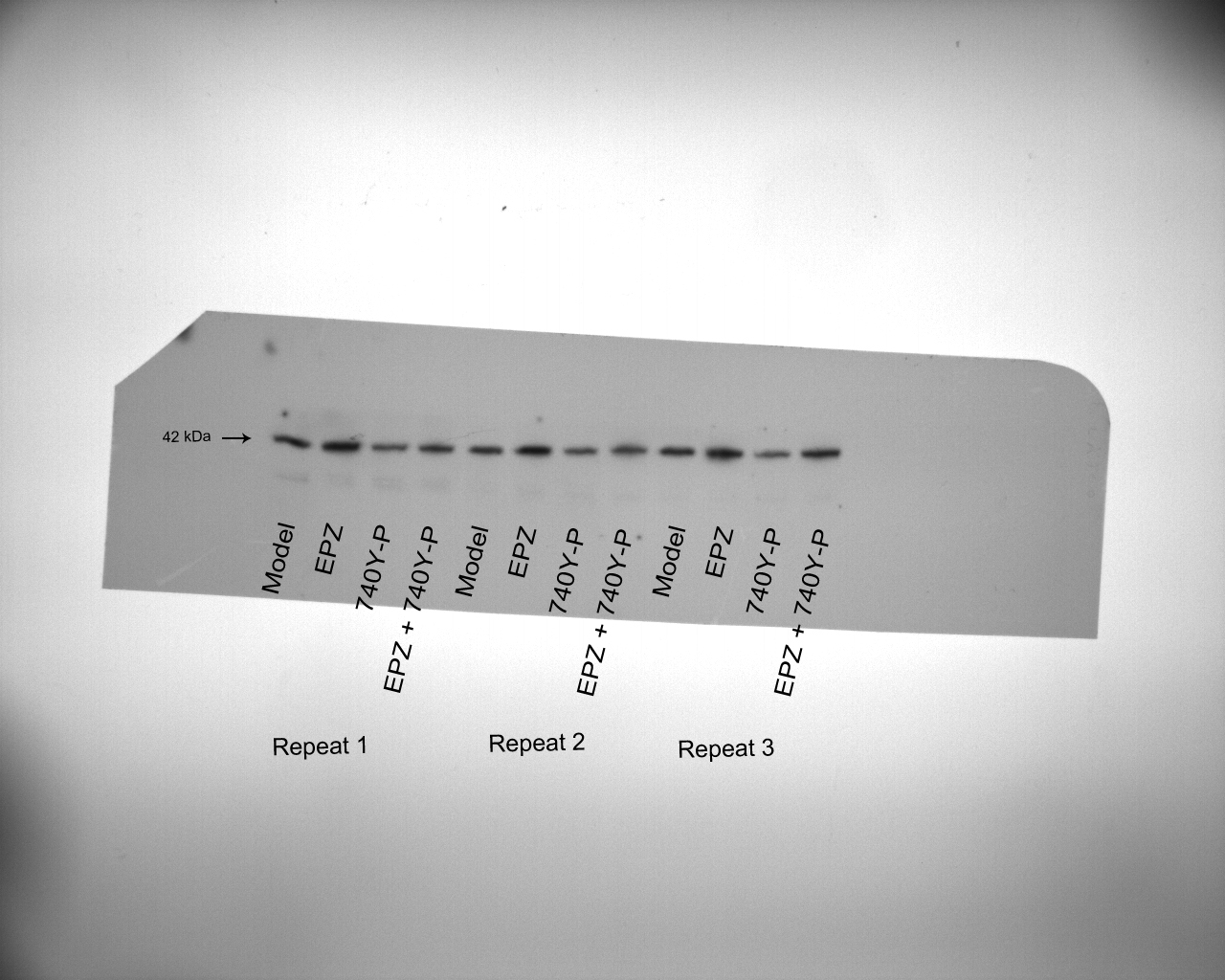

Supplement: Supplementary file 2 [file Data_Sheet_2.ZIP › Uncropped Western Blot Images/Fig5_α-SMA.jpg]

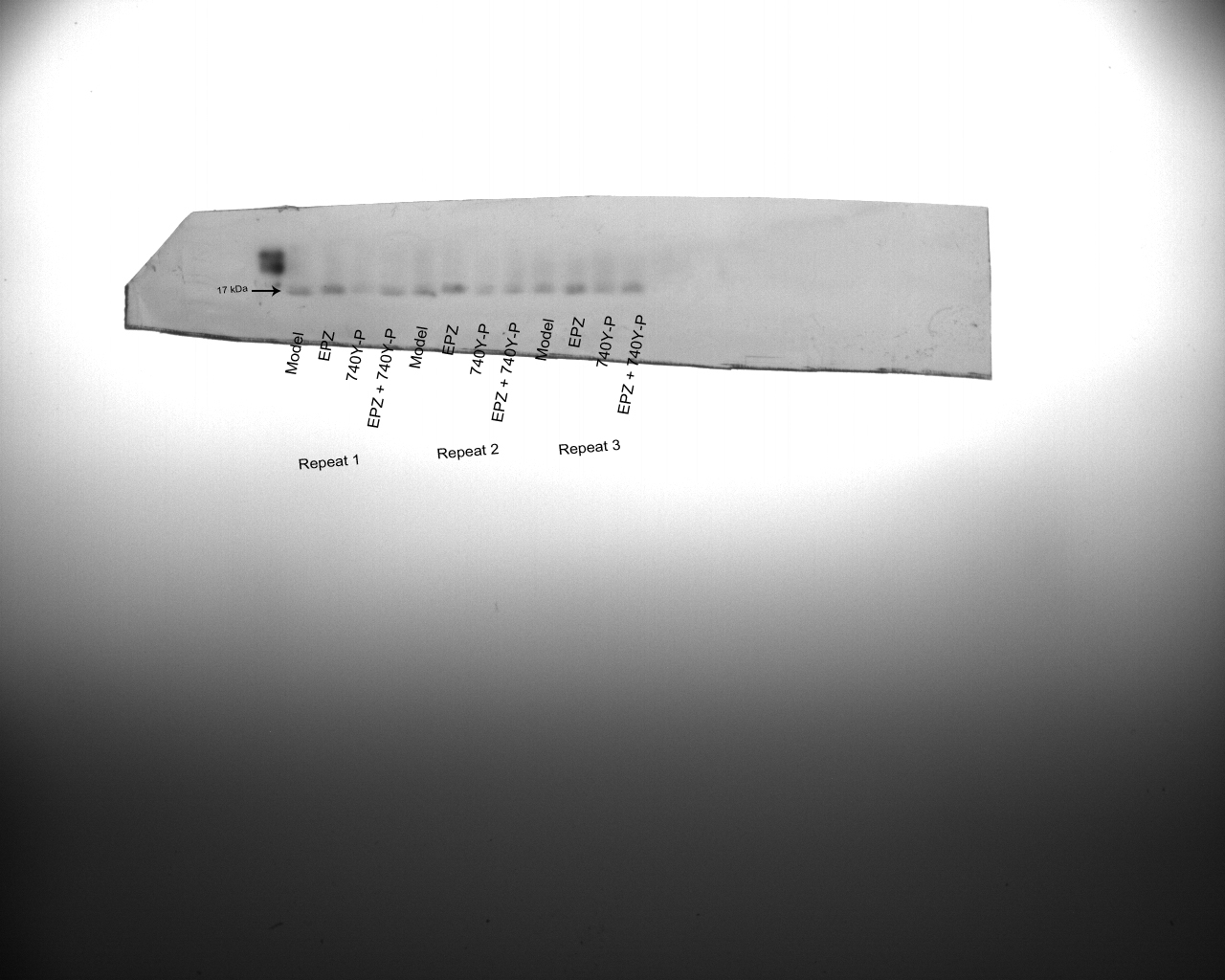

Supplement: Supplementary file 2 [file Data_Sheet_2.ZIP › Uncropped Western Blot Images/FigS2_Cleaved caspase-3.jpg]

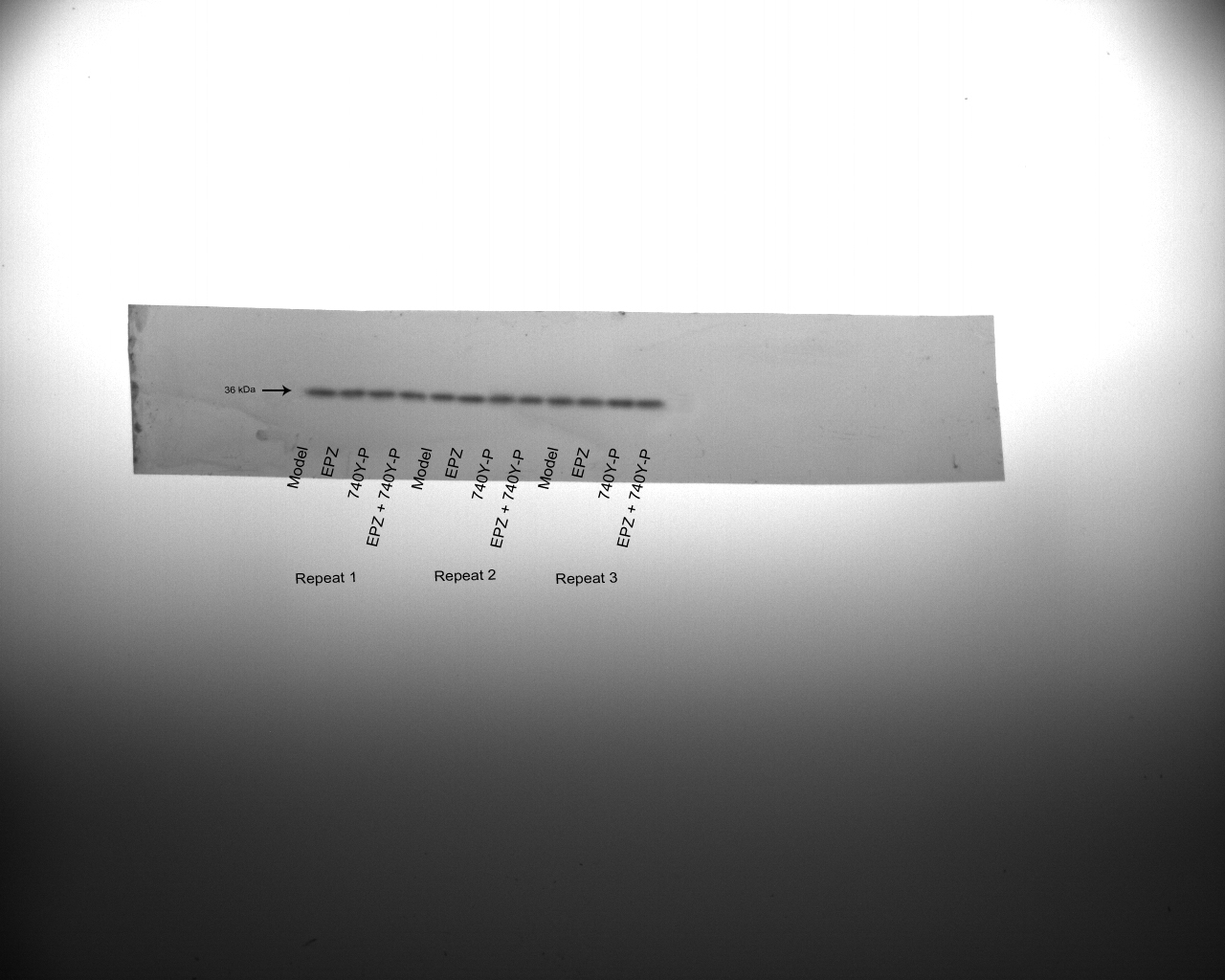

Supplement: Supplementary file 2 [file Data_Sheet_2.ZIP › Uncropped Western Blot Images/FigS2_GAPDH.jpg]

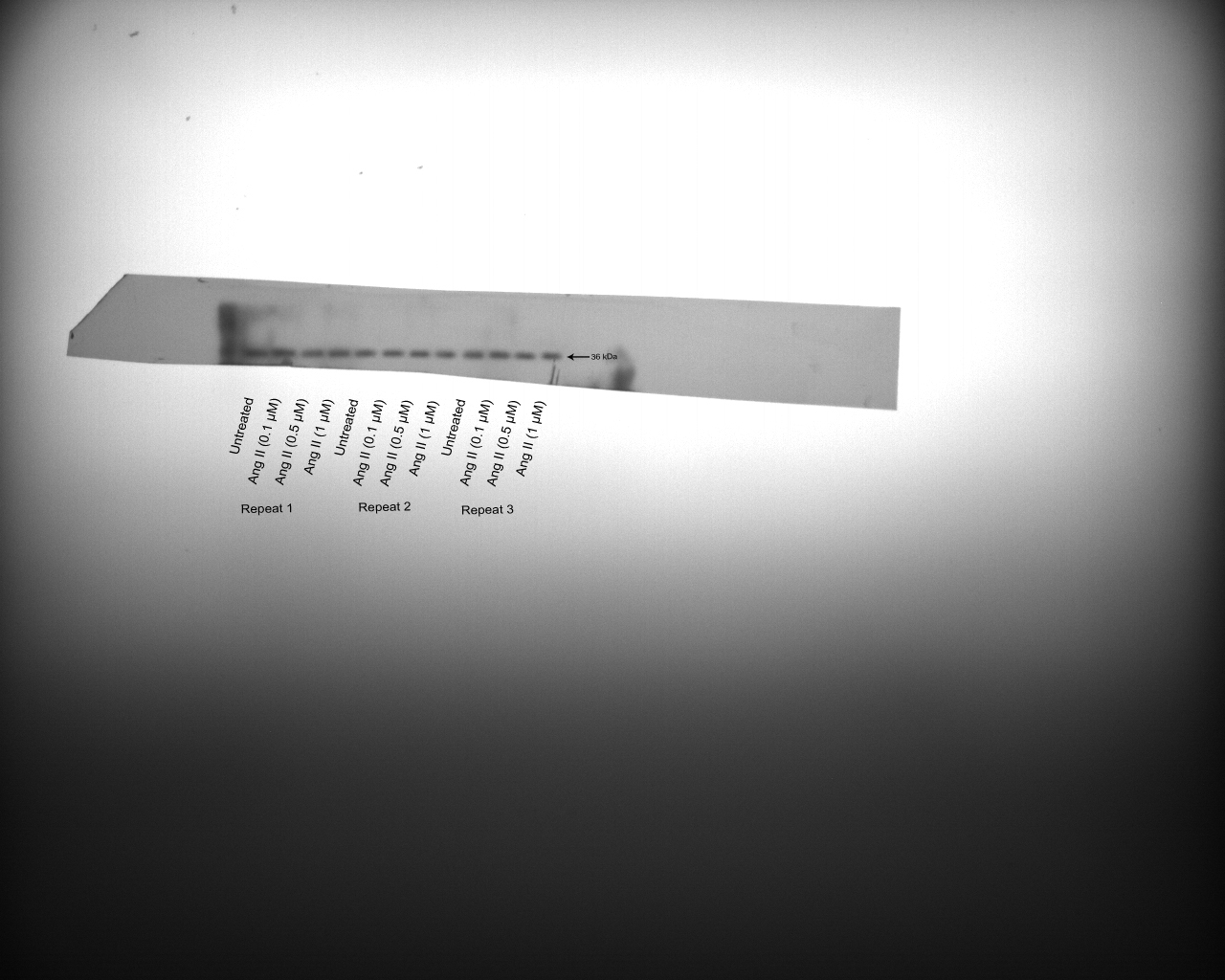

Supplement: Supplementary file 2 [file Data_Sheet_2.ZIP › Uncropped Western Blot Images/FigS3B_GAPDH.jpg]

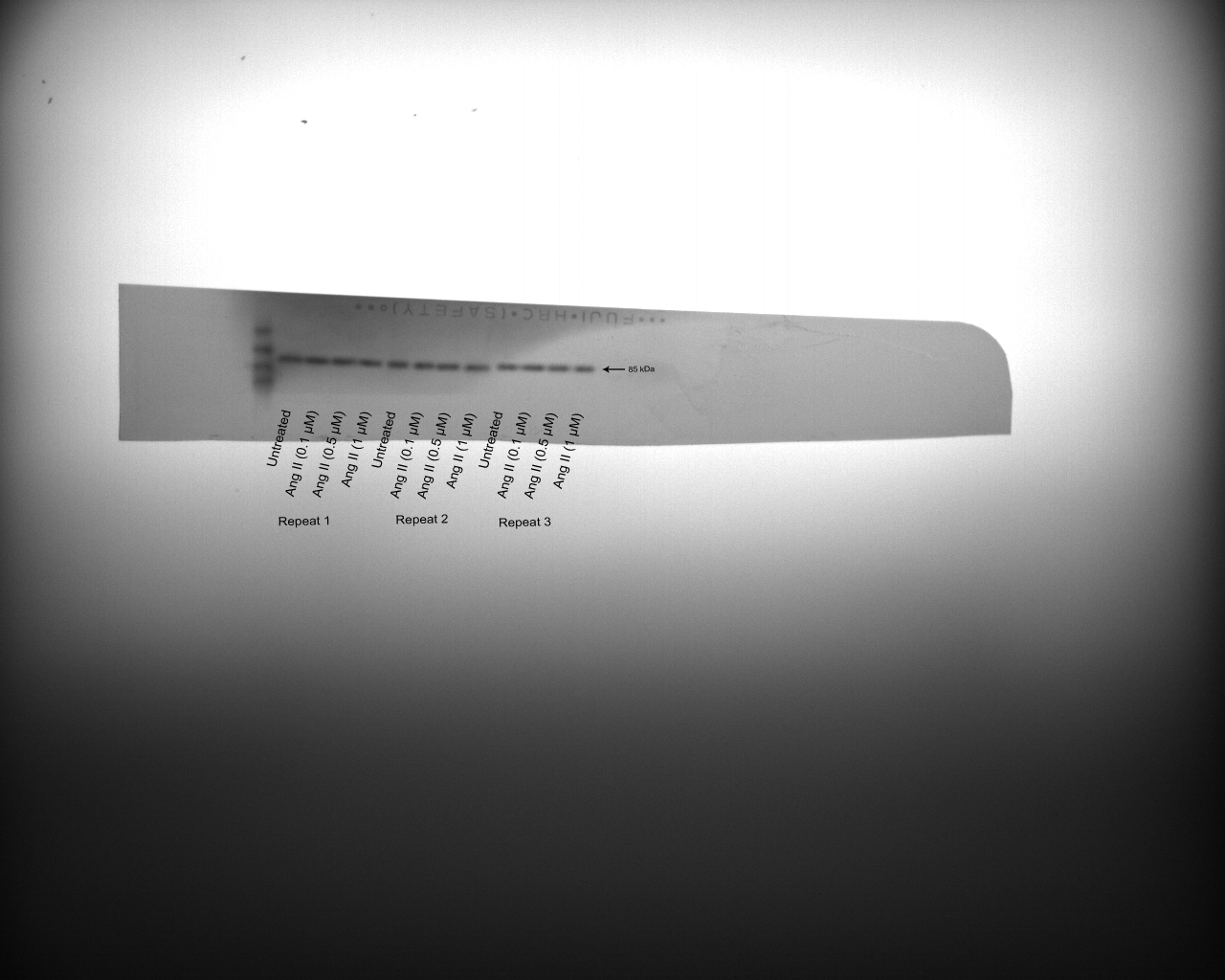

Supplement: Supplementary file 2 [file Data_Sheet_2.ZIP › Uncropped Western Blot Images/FigS3B_PI3K.jpg]

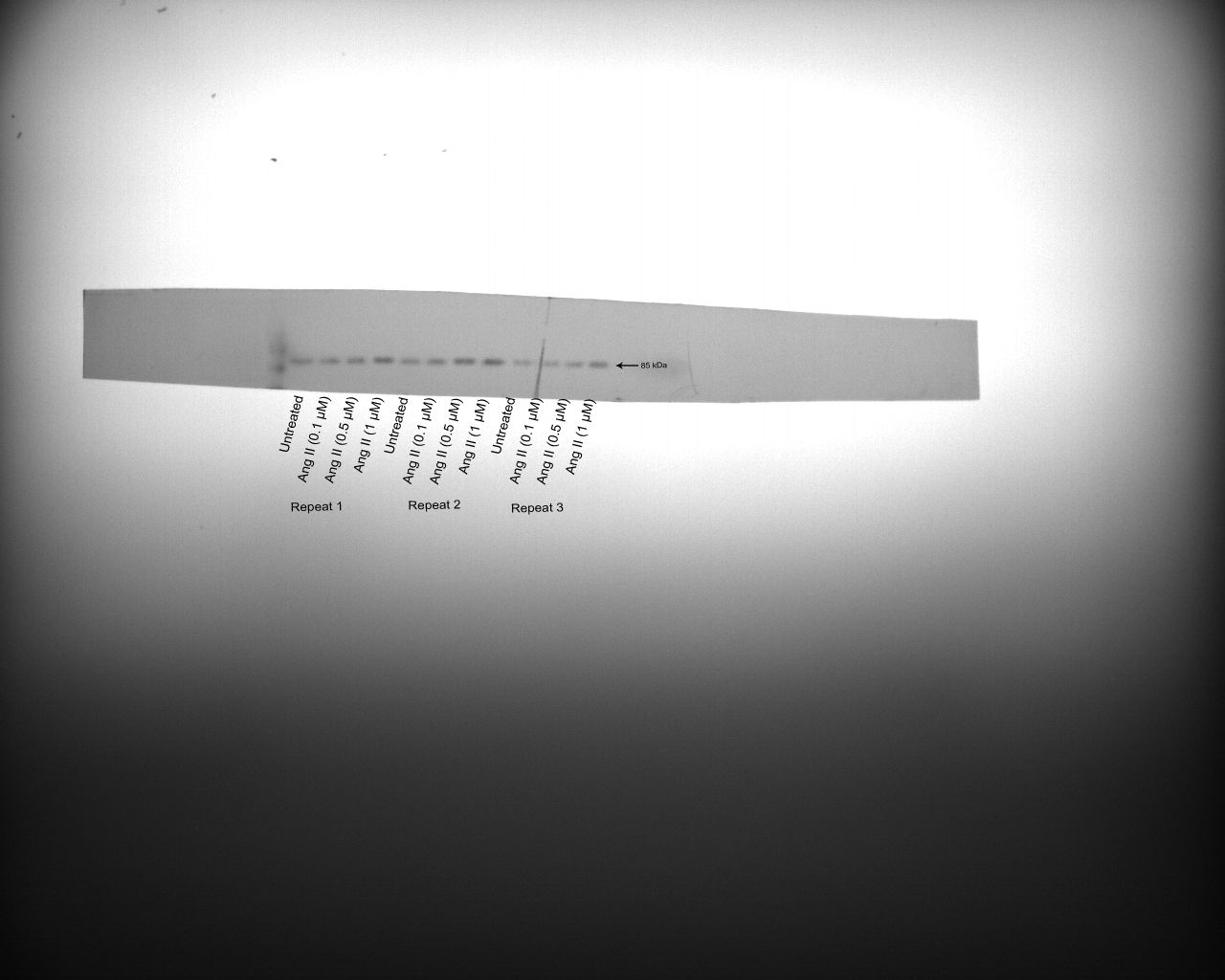

Supplement: Supplementary file 2 [file Data_Sheet_2.ZIP › Uncropped Western Blot Images/FigS3B_p-PI3K.jpg]

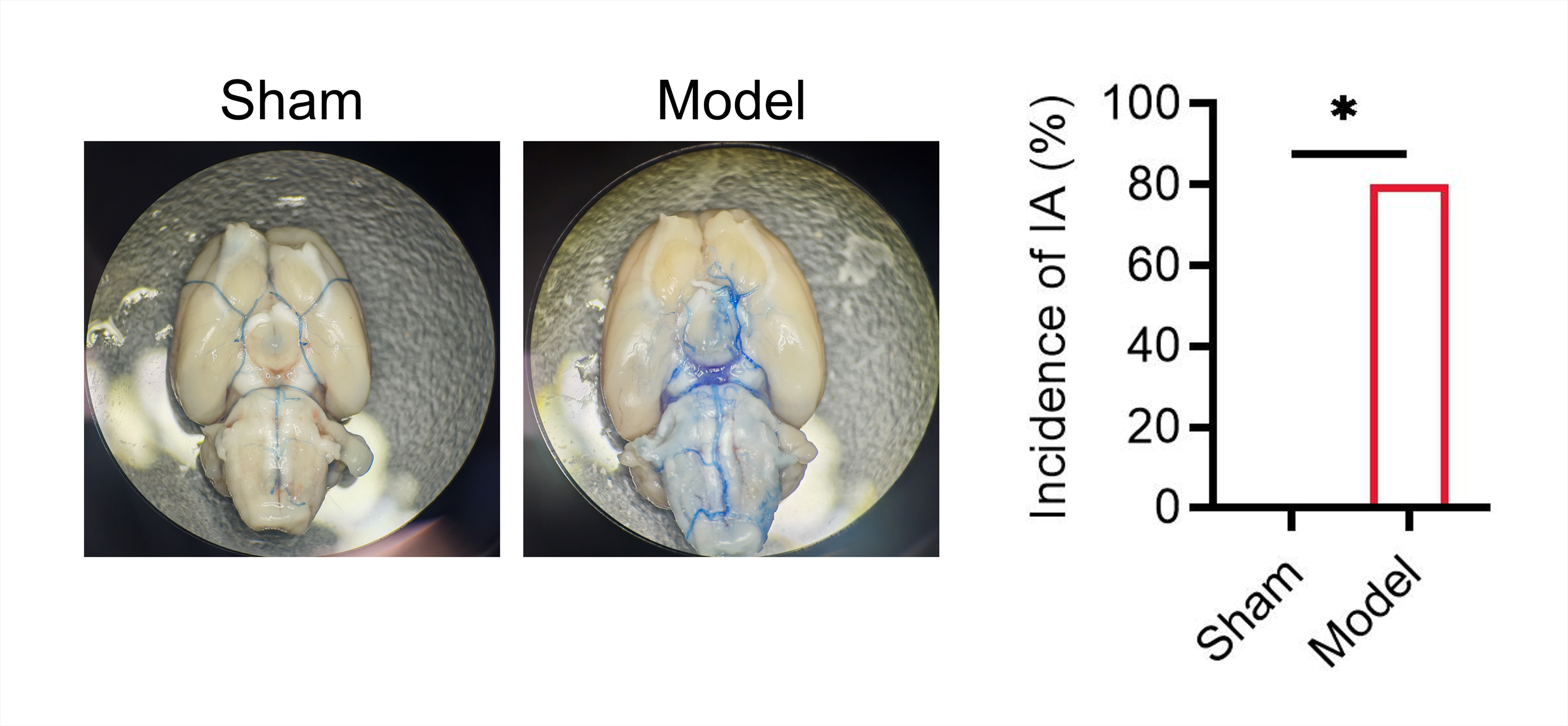

Supplement: SUPPLEMENTARY FIGURE 1 — Confirmation of IA formation. Representative images of cerebral arteries visualized by bromophenol blue-gelatin perfusion and comparison of IA incidence between the Sham and Model groups. *: p < 0.05. [file Image_1.TIF]

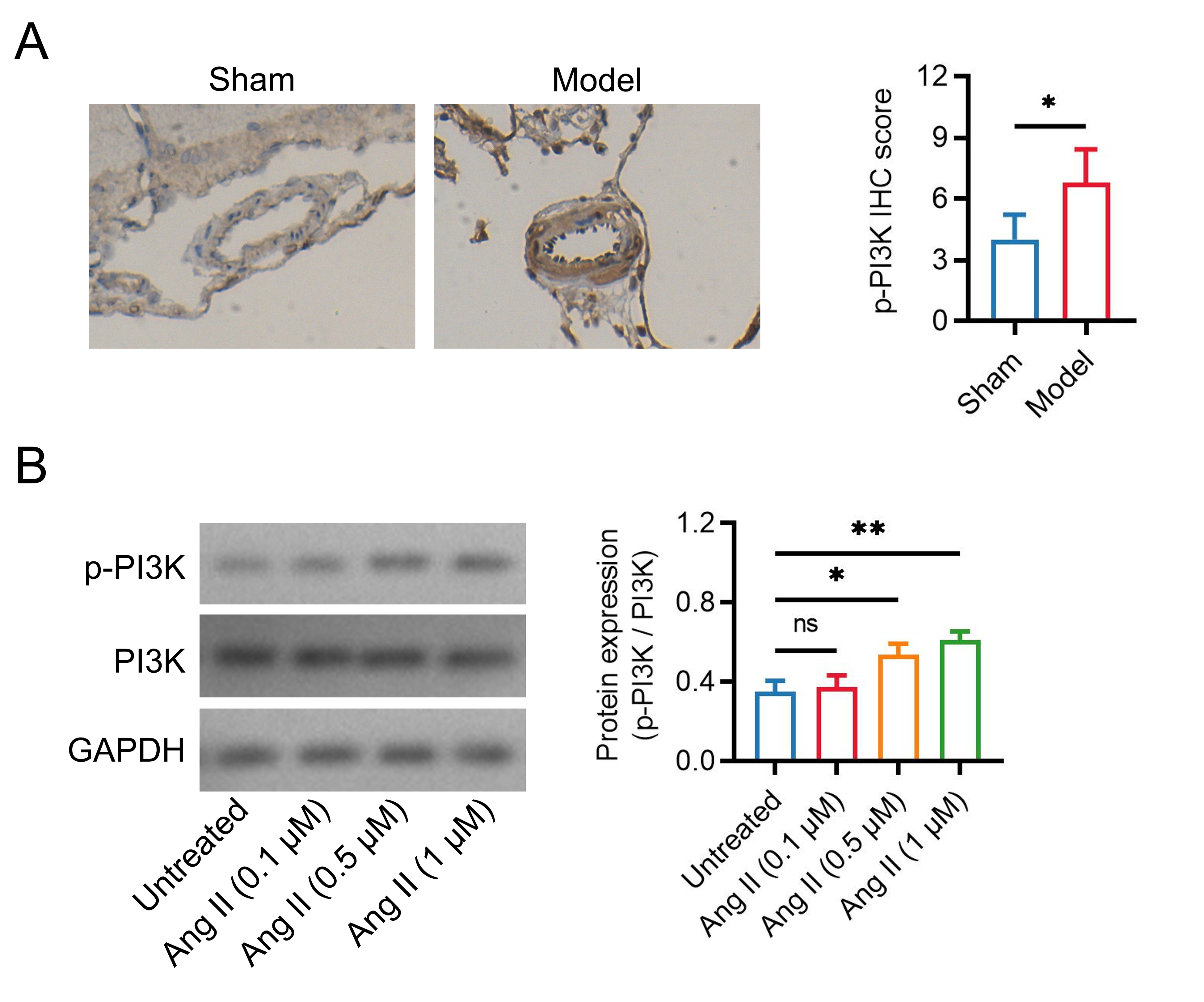

Supplement: SUPPLEMENTARY FIGURE 2 — PI3K level was increased in IA models. In vivo analysis of p-PI3K level between the Sham and Model groups (A). In vitro analysis of p-PI3K level among the Untreated, Ang II (0.1 μM), Ang II (0.5 μM), and Ang II (1 μM) groups (B). *: p < 0.05; **: p < 0.01; ns: not significant. [file Image_2.TIF]

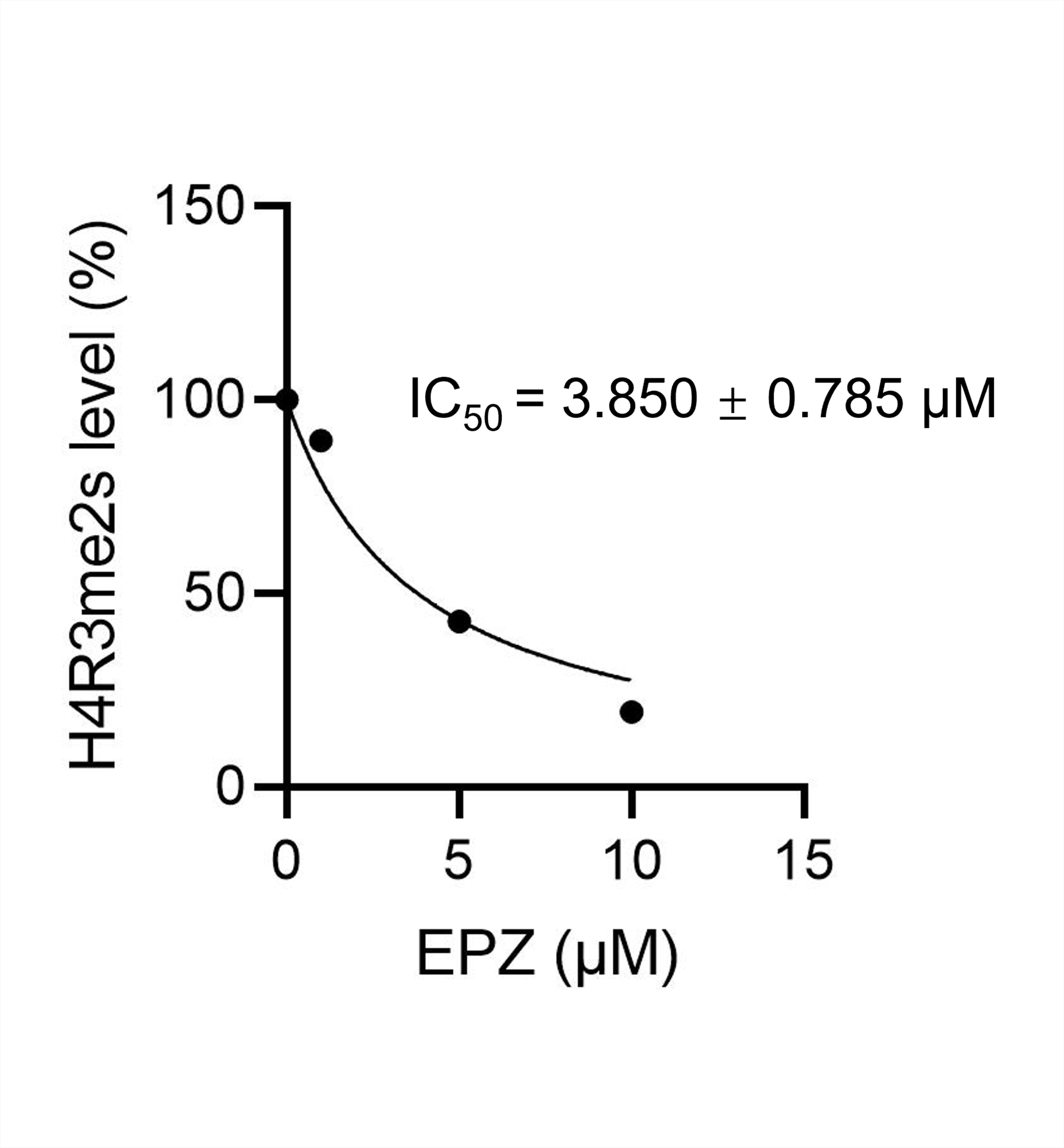

Supplement: SUPPLEMENTARY FIGURE 3 — Dose–response curve of EPZ015666 on H4R3me2s levels. EPZ-induced dose-dependent reduction in H4R3me2s levels (IC50 = 3.850 ± 0.785 μM). [file Image_3.TIF]

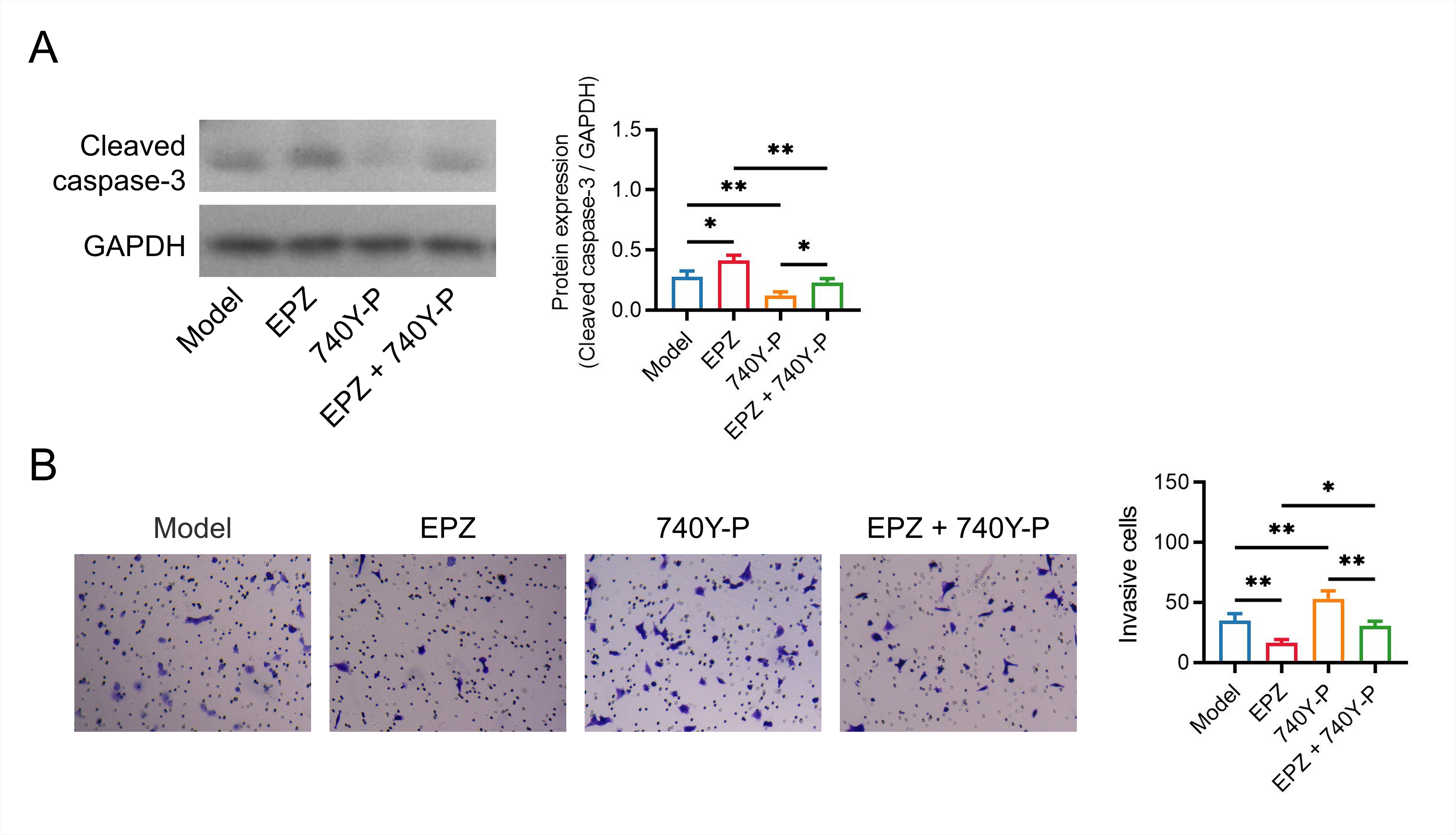

Supplement: SUPPLEMENTARY FIGURE 4 — Confirmation of the effect of EPZ015666 on apoptosis and invasive capacity in Ang II-treated VSMCs. Comparison of cleaved caspase-3 among the Model, EPZ, 740Y-P, and EPZ + 740Y-P groups (A). Comparison of invasive capacity using a shortened incubation time (6 h), distinct from the primary 24 h Transwell assay, among the model, EPZ, 740Y-P, and EPZ + 740Y-P groups (B). *: p < 0.05; **: p < 0.01. [file Image_4.TIF]
